# Are RNA Therapies a Solid Foundation or a Frontier Yet to Be Conquered?

**DOI:** 10.3390/ijms27136086

**Published:** 2026-07-07

**Authors:** Francesco Nappi

**Affiliations:** Department of Cardiac Surgery, Centre Cardiologique du Nord, 93200 Saint-Denis, France; francesconappi2@gmail.com or f.nappi@ccn.fr; Tel.: +33-149334104; Fax: +33-149334119

**Keywords:** microRNA, long non-coding RNAs, non-coding RNAs, ncRNA therapeutics, miRNA biomarker, microRNAs functions, acute coronary syndrome, coronary artery disease

## Abstract

The identification of microRNAs (miRNAs) has resulted in significant advancements in research, particularly regarding their utilization as diagnostic and therapeutic targets. This has generated enthusiasm for exploring the potential of non-coding RNAs (ncRNAs) in treating cancer and other diseases, with miRNAs and long non-coding RNAs (lncRNAs) showing particular promise. Over the past twelve years, there has been significant research into RNA-based treatments. Antisense oligonucleotides and small interfering RNAs are the most commonly used. Certain products have received Federal and Drug Administration approval. Notably, the findings from clinical trials have been inconsistent, with certain investigations indicating notable effectiveness and others reporting only minimal efficacy or safety concerns. Consequently, clinical trials are currently underway to evaluate the efficacy of novel treatment options, including antimiRNAs, in addressing these challenges. There is an increasing interest in the use of long non-coding RNA (lncRNA)-based therapies. The potential for drugs developed using this technology is significant. Significant advancements in preclinical and clinical trials have emerged, indicating promising potential for future developments. MiRNAs are playing an increasingly important role in the diagnosis and prediction of acute coronary syndrome manifestations. Its utilization, whether as a comprehensive approach or in conjunction with existing biomarkers, may be implemented in the foreseeable future, especially in instances of uncertainty regarding diagnosis. The primary objective of this review is to deliver a thorough and detailed assessment of recent progress in the field of microRNA detection and characterization. A key focus of this assessment will be on their clinical translation. Secondly, an exploration of the prevailing knowledge in the field of RNA therapies as potential targets for diagnosis and treatment in the cardiovascular system will be conducted. The most recent challenges and perspectives on the road to clinical application are presented herein. The aim of the present seminar is to furnish a thorough report on the recent advancements in the detection and characterization of miRNAs and lncRNAs, with specific emphasis on their clinical translation. In summary, the paper herein presents an exploration of the most recent challenges and perspectives on the road to clinical application.

## 1. Introduction

**Figure 1:**
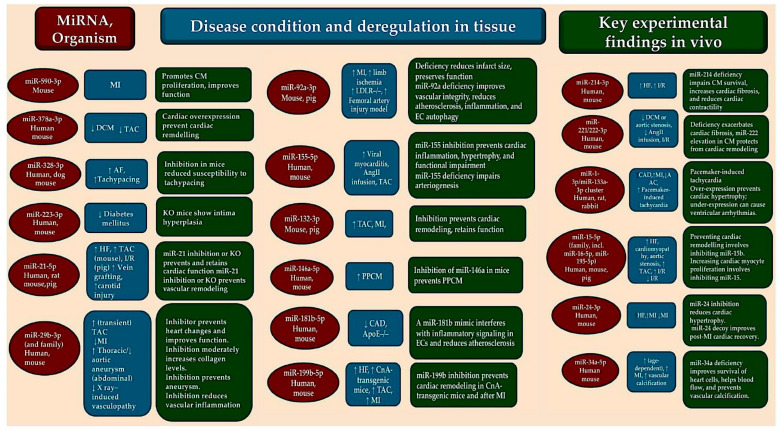
MiRNAs play a crucial role in the cardiovascular system. These genes are associated with various diseases and disease phenotypes, and their effects can be studied in living organisms. The figure illustrates the following: microRNA species and the organism under investigation (dark red), disease state and regulation (blue), and key experimental evidence in vivo (dark green). Abbreviations: AF, atrial fibrillation; AngII, angiotensin II; CAD, coronary artery disease; CnA, human calcineurin subunit A; DCM, dilated cardiomyopathy; HF, heart failure; I/R, cardiac ischaemia–reperfusion; CM, cardiac myocyte; EC, endothelial cell; KO, knockout; SMC, smooth muscle cell; MI; myocardial infarction; PPCM, peripartum cardiomyopathy; AAC/TAC, ascending/transverse aortic constriction. From Nappi et al. Refs. [1,11,12,13,14,15,16,17,18,19,20,21,22,23,24,25,26,27,28,29,30,31,32,33,34,35,36,37,38,39,40,41,42,43,44,45,46,47,48,49]. Down arrow = decrease; Up arrow = increase.

The discovery of small non-coding RNAs in the nematode *Caenorhabditis elegans* three decades ago revealed their function in controlling gene expression post-transcriptionally [1,2,3]. Since then, the identification of numerous microRNAs (miRNA) in higher eukaryotes has demonstrated that these regulate the majority of mammalian miRNAs [4,5,6]. The exact number of miRNAs present in humans is still up for debate. A survey of the 1973 human miRNAs listed in mirBase 22.1 [7] reveals that a significant number of them do not meet strict curation criteria, such as expression, sequence restriction, or evidence of functional ancestral processing. Depending on the source, the number of functional human miRNAs is estimated to be between 556 and 758. However, the number of functionally active miRNAs is further diminished by the fact that most miRNAs only exert their effects at elevated levels of expression in tissues [8,9,10]. To date, approximately 150 miRNAs have been tentatively implicated in the cardiovascular system, although it should be noted that this figure is still subject to change. According to some sources, a more limited number of these, between 30 and 35, have been the focus of substantial research in experimental models and have been confirmed in these settings [11,12,13,14,15,16,17,18,19,20,21,22,23,24,25,26,27,28,29,30,31,32,33,34,35,36,37,38,39,40,41,42,43,44,45,46,47,48,49]. (Figure 1).

Many of these candidates are currently in the next stage of testing, which involves evaluating their side effects in patients. In addition, all significant cancer characteristics examined to date have been associated with elevated or diminished levels of various transcripts, miRNAs, and long non-coding (long nc) RNAs [50,51,52,53,54]. In addition, their participation in numerous biological pathways has been confirmed, including the development and preservation of immune cells, immune system malfunction, neural growth and development, and neurological conditions. As a result of these findings, the strategic targeting of native expressed non-coding ribosomal RNA (ncRNA) has emerged as a promising new therapeutic modality with potential for application in a wide range of disease contexts [55,56,57,58].

A significant number of RNA-based therapeutic interventions have been thoroughly investigated. The following are examples of RNA-based therapeutic agents: antisense oligonucleotides (ASOs), ASO therapeutic circular RNAs (circRNAs), anti-microRNAs (antimiRs), small interfering RNAs (siRNAs), microRNA sponges, microRNA mimics, short hairpin RNAs (shRNAs), and CRISPR-Cas9-based gene editing. For a more thorough analysis of these agents, please refer to the references provided [21,59,60,61].

## 2. Methods

The review was designed and the database investigation was conducted in January 2026. A thorough search was conducted in MEDLINE, Embase and the Cochrane Library using a combination of search terms related to mi RNA, microRNAs, long non-coding RNAs, non-coding RNAs, ncRNA therapeutics, microRNA biomarkers and microRNA functions. These terms were combined with additional search terms, including ‘RNA therapeutics’, ‘Medicines Agency’, ‘Acute coronary syndrome’ and ‘Coronary artery disease’. The selection of publications was focused on recent literature, with an emphasis on those published in the last decade. However, this did not result in the exclusion of highly regarded and widely referenced older publications. In addition, the reference lists of articles identified by this search strategy were thoroughly searched, and relevant articles were selected. It should be noted that the inclusion of publications was restricted to English-language materials, and observational or matched studies that were well-conducted. The quality of the included studies was assessed using the SANRA scale (Scale for the Assessment of Narrative Review Articles) [62], see Table 1.

**Figure 2:**
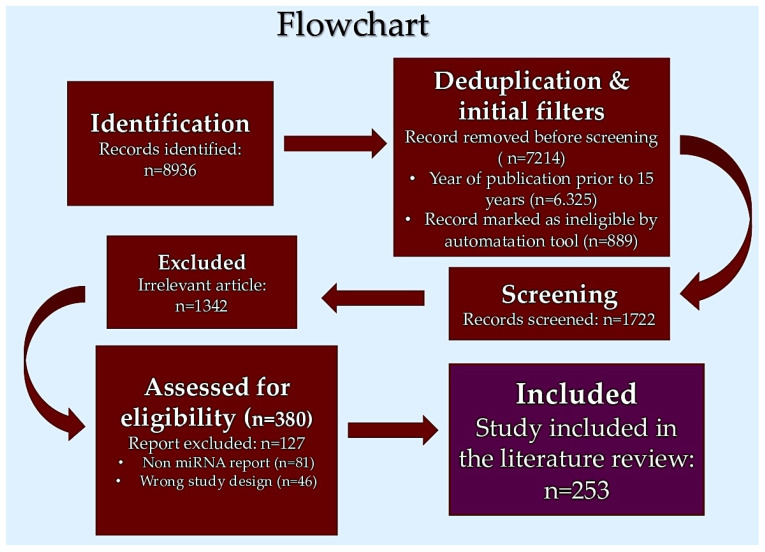
Flowchart.

A comprehensive search of the relevant abstracts was conducted, yielding 8936 results. Following deduplication, 1722 relevant citations were screened. The predefined inclusion criteria were used to guide the review of titles and abstracts (see Table 1). The selected English-language articles comprehensively addressed the subject of miRNAs. These articles also included details that specified the type of microRNA that was studied, as well as the type of animal model or clinical application. Furthermore, particular attention has been given to the results of the international registry, with regard to the number of patients and clinical centers involved in the trial. Following a thorough evaluation of 380 citations, 129 articles were excluded on the grounds that they were not relevant to the subject of microRNA (*n* = 82) or did not meet the necessary study design criteria (n = 47). Consequently, 251 studies were deemed to have met the inclusion criteria and were included in the final narrative review. For a visual representation of the process, please refer to Figure 2, which presents a flowchart.

## 3. Results

### 3.1. Guide to the Types of RNA-Targeted Therapeutics

RNA-targeting therapies are designed to induce functional characteristics analogous to those of microRNAs (miRNAs). The objective of these therapies is to either reverse or reduce the levels of a particular miRNAs or, alternatively, to prevent the binding of a specific miRNAs to its designated target. Chemical modulation is employed to enhance the pharmacokinetics and pharmacodynamics of RNA therapeutics. This is due to their inherent instability and inability to cross cell membranes due to electrostatic repulsion [62,63].

In order to enhance stability, first-generation modifications have been employed to substitute the phosphodiester backbone linkages with phosphothioate (Pt) linkages [64]. An exemplar of a first-generation ASO is Fomivirsen, which is an RNA-based therapeutic that was the first of its kind to be licensed for clinical application (1998). It targets cytomegalovirus (CMV) IE-2 mRNA to treat CMV retinitis. Second-generation derivatives are modified to enhance the bioavailability of the molecules in question. This process increases their efficacy and reduces their toxicity and immunostimulation properties. These objectives can be achieved by substituting the 2′-O-alkyl group of the sugar moiety with, for instance, 2′-O-ME, 2′-MOE, or 2′-F. The following are the possible combinations: 2′-O-ME, 2′-MOE, 2′-MOE, 2′-F, 2′-F, 2′-O-ME, 2′-MOE, 2′-F, 2′-O-MOE, 2′-MOE, or 2′-F [65]. Gapmers are a class of chimeric molecules. They feature a central strand of DNA monomers. This design choice is intended to facilitate RNase H cleavage. This central strand is surrounded by 2′-modified nucleotides, a feature based on the principle that 2′-sugar modifications suppress RNase H activity. The third-generation chemistry process involves altering the furanose ring, leading to the production of locked nucleic acids (LNAs), peptide nucleic acids (PNAs), and phosphoramidate morpholino oligomers (Pmos). All RNA therapeutic agents that are currently approved by regulatory authorities have undergone a chemical adaptation of the second or third generation [66], see Table 2.

Short hairpin RNAs (shRNAs) employ the maturation pathway of miRNAs, which are subsequently cleaved by Dicer to yield a final double-stranded product prior to RNA-induced silencing complexes (RISC) loading. The standard approach for introducing shRNAs into cells has been through the use of viral transfer technologies, which include adenovirus-associated viruses, retroviruses, and lentiviruses. The effectiveness of bifunctional shRNAs in the suppression of gene expression is attributable to their capacity to simultaneously generate transcripts that exhibit both exact and poor complementarity. This phenomenon can lead to a number of adverse outcomes, including degradation and translational silencing [67].

Currently, there are two constructs for delivering bifunctional shRNA molecules by liposomes that are undergoing phase I clinical evaluations. The constructs are referred to as pbi-shRNA eWs/FlI1 and pbi-shRNA eWs/FlI1, respectively. The former targets the microRNA that creates the eWs–FlI1 fusion [68]. The present text aims to provide an examination of the clinical trials NCt02736565 and NCt01505153. The objective of this investigation is to determine the effectiveness of the protein in treating Ewing’s sarcoma and pbi-stmN1, respectively, with regard to targeting stathmin 1 microRNA in advanced solid tumors [69]. MiRNA mimics leverage the capacity of endogenous miRNAs to address multiple mRNAs concurrently. The structural similarity between mimics and their endogenous counterparts is notable, with the passenger chain featuring a reduced number of mismatches. This prevents RISC loading and averting potential action as anti-microRNA (antimiR) [70]. Two microRNA mimics, mRX34 and mesomiR-1, have been the subject of clinical investigation as a potential therapeutic intervention for cancer treatment. Research has shown that mRX34 mimics the effects of a specific microRNA called miR-34 [71,72], while mesomiR-1 has been demonstrated to mimic the actions of a distinct miR-16 [73]. see Table 3.

AntimiRs represent a class of therapeutic agents, specifically formulated as antisense oligonucleotides (ASOs). These agents are designed to be either completely complementary or selectively complementary to a specific endogenous microRNA. This strategic design aims to hinder the interaction between the microRNA and its target genes, thereby modulating biological processes that are critical in disease progression and regression. AntimiRs, when conjugated with cholesterol to enhance their intracellular delivery, are alternatively referred to as ‘antagomiRs.’ Two examples of antagomiRs that have entered clinical trials as novel hepatitis C virus (HCV) therapeutics are Rg-101 (N-acetylgalactosamine-conjugated ASO) (Table 3) and miravirsen (sPC3649; beta-D-oxy-lNA) [74,75]. Additionally, an antimiR-92a compound, designated as mRg-110, was the subject of a phase I/II trial to evaluate its capacity to selectively stimulate angiogenesis and enhance wound healing (NCT 03603431). Furthermore, the investigation centered on the efficacy of aantimiR21 [Rg-012] in mitigating kidney fibrosis in patients diagnosed with Alport syndrome (NCT 03373786).

MiRNA sponges represent a type of non-coding RNA molecule that has been specially designed to contain multiple binding sites for various microRNAs. This unique attribute allows them to selectively capture and sequester these microRNAs [76,77,78,79]. This technology has been demonstrated to be effective in targeting both individual microRNAs, including mir-21, miR-155, and miR-221/miR-222, as well as entire seed families, such as miR-181a, miR-181b, and miR-181c, within tumor cells. It is possible to target one or more miRNAs, such as mir-21, miR-155, and miR-221/miR-222, in tumor cells [80,81]. It has been demonstrated that RNA-based sponges have proven effective as a research instrument [82]. However, their translation into the clinical domain remains to be achieved [83].

MiRNA-masking ASOs represent a gene-specific and safe therapeutic strategy, whereby the binding site of a microRNA within the target gene is masked [84]. This approach is especially relevant in instances where family members demonstrate dual roles [84]. Additionally, small 8–10 nt lNAs can be used to specifically silence seed sequences [84]. In addition, the use of 8–10 nt lNAs has been shown to be an effective method for silencing specific seed sequences [85]. The restoration of tumor-suppressing functions of microRNA (miRNA)-16 in melanoma cells was achieved by masking the binding sites of this RNA within the 5′ UTR of the transferrin receptor protein-coding mRNA. This was accomplished using an 16-nt oligonucleotide. This process, enabled by the noncanonical nature of the miR-16 binding sites, has been shown to effectively neutralize the tumor-suppressing function of miR-16 within the context of melanoma cells [86]. However, this methodology is not yet widely employed in clinical practice, as the application of locked nucleic acid (LNA)-masked antisense oligonucleotides in clinical settings remains in its infancy.

There has been a significant increase in research focusing on the potential of non-coding RNA (lncRNA) therapeutic development in recent years. However, there are currently no clinically viable therapeutic applications of these molecules that have been translated. The primary investigative focus at present is to explore the utility of these molecules as diagnostic tools, owing to their association with various pathological conditions, including preeclampsia (NCT 03903393), lung cancer (NCT 03830619), and acute ischemic stroke (NCT 04175691). The expansion of the present set of ncRNAs has the potential to increase the number of potential RNA interference and CRISPR targets. Furthermore, it is predicted that certain types of ncRNAs, such as circular RNAs and natural antisense transcripts, will offer exciting new treatment options in the future.

The FDA, in collaboration with the European Medicines Agency (EMA), has approved a total of 13 RNA-based therapeutic compounds that target gene alterations in the liver, muscle, and central nervous system (Table 2). The majority of these medications are either siRNA or antisense oligonucleotides (ASOs). These medications function by either targeting specific genes for downregulation or by disrupting the process of pre-mRNA splicing. This disruption can result in exon skipping or inclusion.

A number of RNA-based therapeutic compounds, including more recent formulations such as miRNAs mimics and antimiRs, are currently undergoing phase II or III clinical evaluation. However, to date, there have been no clinical trial applications in the field of therapeutic intervention using the lncRNA. Table 4.

MiRNA-based therapeutics have the potential to offer two distinct benefits [87,88,89]. First, unlike man-made chemotherapeutic agents or ASOs, miRNAs are naturally expressed substances in human cells. This inherent characteristic ensures that the necessary machinery for their production and targeted delivery is already present in the cells. Secondly, it has been demonstrated that microRNAs function by interfering with multiple genes that are involved in a single pathway, thereby eliciting a response that is both broad and specific. The miR-15–miR-16 cluster offers a prime example of this phenomenon, demonstrating the potential of a single molecule to exert a multifaceted impact on a specific cancer attribute by modulating multiple anti-apoptotic factors, such as BCL-2 and MCL1. As indicated by Calin et al. and Cimmino et al. [90,91], naturally arising miRNAs offer a potentially promising alternative to current RNA-based therapeutic modalities, with the ability to enhance therapeutic efficacy beyond that of synthetic siRNAs or ASO, which are limited to a single targeted molecule.

Long non-coding RNAs have a wide range of functions, making them promising candidates for targeted therapeutic interventions. The approach to targeting these non-coding RNAs should be customized based on their distinct modes of action. There are several methodologies available for targeting these non-coding RNA molecules. These include the inhibition of transcription or post-transcription, the hindering of secondary structure formation or protein interactions, the introduction of synthetic long non-coding RNAs (e.g., circular), and the modification of long non-coding RNA genomic loci or modes of expression using CRISPR-Cas9 or CRISPR-Cas13 [92].

The study of natural antisense transcripts (NATs) has emerged as a fascinating field of scientific inquiry. Long non-coding RNAs are a class of molecules that are transcribed in the antisense (opposite) direction to the genes they encode. This process has a negative impact on their function and is known as “cis-acting”. Preclinical studies have shown the promising potential of ASO treatment in targeting natural antigens (NATs) for stimulating gene reactivation within the central nervous system (CNS). AntagoNATs have been shown to effectively enhance levels of brain-derived neurotrophic factor (BDNF), a protein that plays a central role in memory development [93].

Furthermore, AntagoNATs have been observed to increase the expression of the healthy allele of the SCN1A gene, which has been linked to Dravet syndrome [94]. Specifically, a minimally invasive nasal depot (MIND) has been used to successfully administer antagoNATs that target BDNF-AS through the blood–brain barrier in a murine model. MIND has been shown to successfully navigate the olfactory submucosal space and has been demonstrated to have an approximate 40% success rate when compared with more invasive administration methods [95]. These encouraging results indicate that lincRNA-based therapeutic interventions are on the verge of entering clinical trials.

The clinical translation of RNA-based therapeutics is hindered by challenges related to specificity, release, and tolerance. Issues of specificity may arise from unintended on-target effects in non-target cells or off-target effects from sequence variations or overdosing beyond endogenous levels. The delivery of RNA constructions poses significant challenges due to three fundamental issues: the inherent instability of unmodified RNA; the necessity for endosomal escape mechanisms to facilitate effective intracellular delivery; and the absence of a compatible delivery system for the targeted organ/cell type. In addition to the previously mentioned concerns, clinical trials are often discontinued due to suboptimal outcomes as shown in Table 3.

Clinical trials are often discontinued due to suboptimal outcomes. For instance, Genasense (G3139), a nuclease-resistant ASO that targets BCL2 mRNA, was discontinued due to its inefficacy [96]. This stands in contrast to the highly promising application of venetoclax, a small molecule that mediates the inhibition of the BCL-2 protein [97,98]. The issue of tolerability is related to the recognition of RNA structures by pathogen-associated molecular pattern (PAMP) receptors, such as Toll-like receptors (TLRs). This can result in undesired immune responses. For instance, the microRNA mimic MRX34 has been associated with severe adverse effects in five patients, including cytokine release syndrome. This observation was made during a multicenter phase I clinical trial in subjects with clinically proven progressive malignancies [71,72].

Conversely, the restoration therapy of miRNAs-16 in patients with mesothelioma has been shown to be effective (MesomiR-1), while the management of keloid scars through the intradermal injection of a miRNAs-29 mimic called Remlarsen has been demonstrated to be safe. Initial studies using cobomarsen, which acts as an inhibitor of microRNA-155, in cutaneous T-cell lymphoma, also do not appear to be associated with life-threatening toxicities [59,60]. It can be concluded that, with appropriate toxicological assessments and advancements in delivery methods, the potential for therapeutic applications of microRNAs is a viable proposition.

To improve our understanding of the true role of non-coding RNA in therapeutic interventions, it is essential to explore methods that can overcome the challenges faced when using RNA-based therapeutic agents in the clinical setting. The challenges posed by immune responses, low specificity, and nonspecific delivery, with a particular focus on miRNAs and long non-coding RNA (lncRNA)-based therapeutics, represent another point of concern that must be elucidated. It should be noted that RNA-based interventions have the potential to treat diseases caused by pathogenic RNAs, including those derived from the human genome and xenogenomes. Such diseases include RNA viruses, such as SARS-CoV-2. This comprehensive seminar is intended to offer a detailed overview of the latest and most promising advancements in preclinical and clinical research in this field [98,99].

### 3.2. MicroRNA Initiates with Biogenesis, Stability, and Strand Targeting

**Figure 3:**
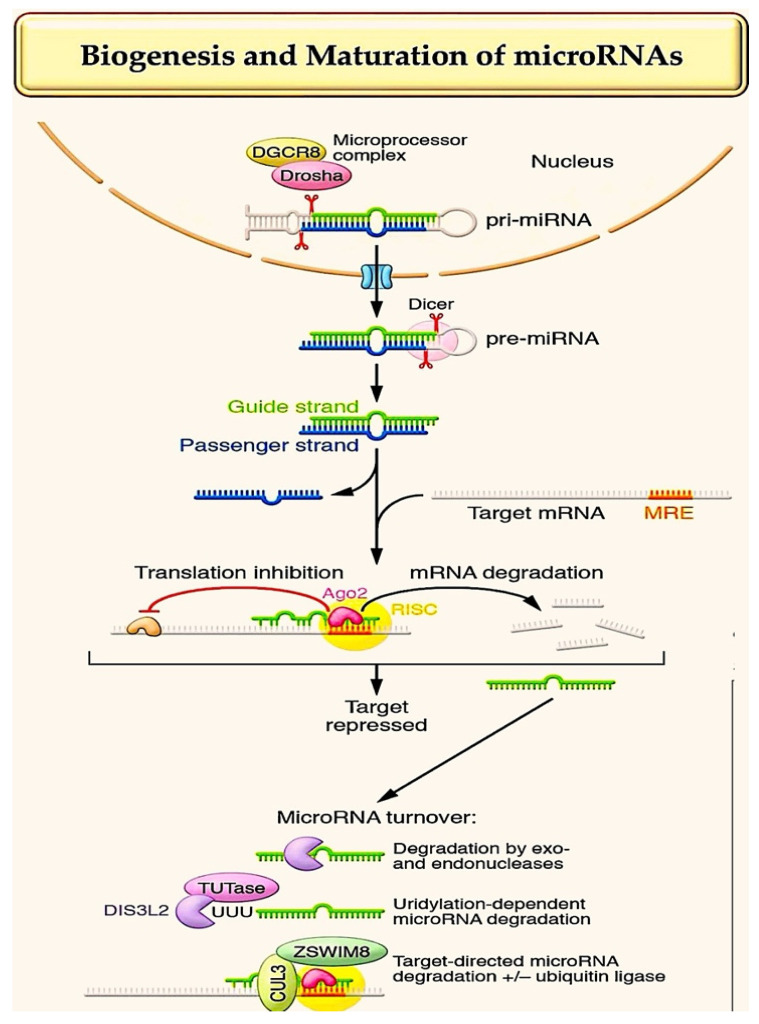
This figure illustrates the biogenesis and operation of microRNAs. The overall process can be categorized into three primary stages. The initial process involves the synthesis and release of nuclear pre-miRNAs into the cytoplasm, where the final synthesis of activated RNAs is promoted in parallel with the production of RNA duplexes and RISCs, as well as RNAi. The following aspects have been reported: the canonical elaboration, functional activation, mechanism of action, and degradation pathways of miRNAs. The process of canonical miRNA biogenesis begins with the formation of larger hairpin RNA molecules. These larger molecules are termed “pre-miRNAs”. These are produced by RNA Pol II transcription of miRNA genes or clusters, or occur as part of introns. The next step in the process is the cleavage of these molecules by a microprocessor complex. This complex comprises the endonuclease Drosha, the DGCR8 protein, and other factors. Abbreviations; DGCR8, DiGeorge critical region 8 protein; DIS3L2, DIS3 like 3′–5′ exoribonuclease 2; miRNA, microRNA; miRNA duplex, precursor miRNA; RISC complex, RNA-induced silencing complex; RNAi, RNA activation; TDMD, target-directed microRNA degradation; TUTases, terminal uridyltransferases. Ref. [1].

Transcripts that regulate gene expression and protein function are known as non-coding ribonucleic acids (ncRNAs). These are derived from the non-protein coding part of the genome. There are two major families of ncRNAs: microRNAs (miRNAs) and long non-coding RNAs (lncRNAs). Figure 3 is intended to provide a visual representation of the processes involved in the formation and maturation of microRNAs [100,101,102].

The process of synthesizing long primary miRNA transcripts is catalyzed by RNA polymerases II and III. The nuclear ribonuclease Drosha and DgCR8 then process these transcripts, resulting in the development of a 70 nucleoside-based transcript that possesses a stem-loop secondary structure. It is a widely held view among scientific professionals that pre-miRNAs are exported from the nucleus by exportin 5 and RangtPase. Subsequently, the RNase III enzyme Dicer cleaves the pre-miRNAs, yielding a mature, double-stranded RNA molecule that is known as a microRNA. Following this process, the resulting duplexes will measure 21–22 nucleotides. One strand, hereafter referred to as the guide strand, will then combine with the RNA-induced silencing complex. It has been demonstrated that a second strand, classified as the ‘passenger’ or ‘driver’ strand, exhibits a tendency to incorporate into the RISC or undergo degradation at a more rapidly accelerated rate [103,104].

It is critical to understand that the process of gene regulation is achieved through the degradation of the microRNA molecule by the action of an RNA helicase. This process subsequently results in the integration of the guide strand of the miRNA into the RISC. Post-transcriptional gene silencing is achieved by the binding of miRNAs to a nucleotide complement of the 3′UTR, 5′UTR, or coding region of a target miRNA. The 5′ end of a microRNA contains a “seed sequence” of nucleotides 2 to 7. In the event of a perfect binding of the seed sequence with its complement, the miRNA it targets is degraded and deadenylated. In cases where binding is imperfect, as it is more often, translational inhibition ensues. The process of both these phenomena is facilitated by RISC [100,101,102,103,104].

In the event of the preservation of both strands, their ability to perform discrete functions becomes evident. This phenomenon has been demonstrated for cardiovascular miR-21 and miR-126 [105,106]. Furthermore, there are microRNA strands that localize to the nucleus and function in unusual ways [106,107]. MiRNA is bound to Argonaute 2 (AGO2) endonuclease and other proteins within RISCs, which are able to regulate both siRNA and miRNA. The requirement for mammalian miRNAs is limited to a seed sequence of seven to eight nucleotides near the 5′ end, exhibiting only full target complementarity, in contrast to the more stringent criteria exhibited by siRNAs, which demand a complete match to their target sequence [102,108]. However, it is important to note that only a limited number of microRNAs (miRNAs) are contingent on these interactions, as evidenced in the extant literature [102,108,109].

Nonetheless, it is hypothesized that additional pairing beyond the seed sequence may facilitate target detection. MicroRNA response elements (MREs), also known as microRNA target sites, have been shown to be predominantly located within the 3′-untranslated region (UTR) of miRNAs, with less frequent occurrence in the 5′-UTR or coding regions. As stated in the research by Grimson et al. [6], and Bartel et al. [102], miRNAs can be categorized into two primary groups based on their functional characteristics. The first group is distinguished by its predominant role in inducing degradation, while the second group is characterized by its function in translational silencing of target mRNAs [102]. Non-genetic miRNA variations, termed isomiRs, resulting from alternative processing, addition of nucleotides or editing of miRNAs [110,111,112], further enrich the miRNA repertoire. A variety of cardiovascular isomiRs has been identified. The expression levels of these isomiRs vary across different pathological conditions. As illustrated by Matsui et al. [113], the levels of these isomiRs exhibit variation in different diseases, implying a possible involvement in pathological processes.

Distinct variant and template targetomes were identified for isomiRs miR-487b-3p and miR-411-5p [114,115,116]. The cessation of a miRNA’s function leads to its enzymatic degradation. Studies have shown that miRNAs have a longer half-life than isomiRs. However, the enzymatic degradation of miRNAs can vary depending on factors such as the miRNA strand and sequence, the specific cell type, and the presence of trans-acting factors. As outlined by Kingston et al. [117] and Marzi et al. [118], the enzymatic degradation of microRNAs is a dynamic process that can vary significantly between different microRNA variants. (Figure 2). The targets of microRNAs represent an additional factor to be considered. Despite the mechanistic details of target-directed microRNA degradation (TDMD) having been elucidated [52,119,120] and its significance having been demonstrated [121], the identification of mRNAs that are involved in TDMD remains difficult.

According to the most recent research, long non-coding RNAs are defined as transcripts of a greater size, with a minimum length of 200 nucleotides. These molecules are transcribed similarly to mRNAs, but they do not undergo processing to be translated into proteins [122]. The field has identified two primary classifications of functional components within this group of molecules. The first of these is the interactor component, which has been found to engage in direct physical interactions with other nucleic acids, proteins, or lipids.

The second is the structural component, the role of which is to give rise to secondary and/or tertiary 3D RNA structures, thus regulating specific functional relationships [123].

Long non-coding RNAs have the capacity to interact with DNA, RNA, and proteins through base pairing in linear form or chemical interactions in secondary structures. This capacity confers upon them the potential to function in a more varied manner than that of miRNAs. A considerable number of identified lncRNAs have been demonstrated to serve a regulatory function in gene expression, exerting influence on factors such as transcription factor activity and epigenetics. Furthermore, interactions with mRNAs have been demonstrated to affect their stability or rate of translation. Furthermore, research indicates that interactions between long non-coding RNA and proteins influence their stability, activity, and subcellular localization [124,125]. Additionally, circular RNAs, which are structurally comparable to lncRNAs, have been demonstrated to act as competitive endogenous RNA (ceRNA) sponges, thus modulating miRNA activity [126,127].

### 3.3. MicroRNA-Targeting Therapy Is Advancing Towards Cardiovascular Disease

**Figure 4:**
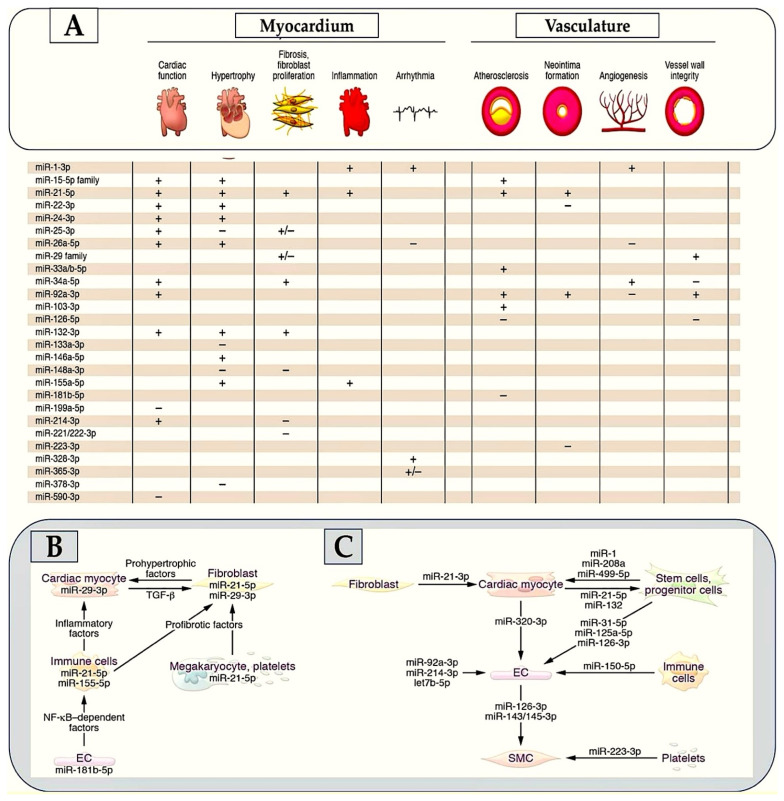
The function of miRNAs in the cardiovascular system is as follows: (**A**) offers a concise overview of the role that microRNAs play in the context of heart muscle and blood vessels. A positive sign (+) indicates that the miRNA promotes the specified process, while a negative sign (−), conversely, indicates that the miRNA prevents it. The subsequent chapters offer comprehensive insights into these molecules, detailing their role in regulating cardiac function, with particular emphasis on the consequences of altered levels. (**B**) offers a thorough review of the various miRNAs that play critical roles in regulating targets associated with intercellular communication within the cardiovascular system. (**C**) details the specific paracrine functions of certain miRNAs within the cardiovascular system. Conversely, the release of the miR-21 core fragment by endometrial mesenchymal stem cells has been observed to engender cardioprotective effects by promoting cell survival and angiogenesis, according to research findings. In addition, studies have shown that certain miRNAs present in the myocardium can stimulate the mobilization of progenitor cells in the bone marrow. Platelets carry microRNA-223-3p, a regulatory molecule that plays a key role in the differentiation and proliferation of vascular SMCs. Abbreviations: EC; endothelial cell; miRNA, microRNA; SMC, smooth muscle cell. Refs. [1,12,13,14,15,16,17,18,19,20,21,22,23,24,25,26,27,28,29,30,31,32,33,34,35,36,37,38,39,40,41,42,43,44,45,46,47,48,49,100,105,120,128,129,130,131,132].

As demonstrated in Figure 1, 30 to 35 miRNAs have been identified as playing critical roles in cardiovascular health, with strong evidence supporting this assertion. Figure 4 provides a visual representation of the processes involved in the biogenesis and maturation of miRNA. When these miRNAs are subjected to manipulation, they have been observed to induce discrete pathophysiological effects within the myocardium or vasculature, as illustrated in Figure 4A. The activation of signaling pathways that result in the secretion of protein factors is a feature of some of these (Figure 4B), while others are components of extracellular vesicles, specifically of the exosome variety (Figure 4C). It is evident from the expansion of knowledge in the field and the increased use of miRNAs as therapeutic agents in the myocardium and vasculature, as illustrated in Figure 4, that this research is of significant value and is likely to continue to be highly relevant in future studies [12,13,14,15,16,17,18,19,20,21,22,23,24,25,26,27,28,29,30,31,32,33,34,35,36,37,38,39,40,41,42,43,44,45,46,47,48,49,100,105,119,120,128,129,130,131,132].

#### 3.3.1. Roles of MicroRNAs in the Cardiovascular System

Research has shown a significant increase in the expression of microRNA-21-5p in human cardiac muscle that has been compromised. The association of this microRNA species with fibrosis is well documented. In particular, the results indicate that miR-21-5p is overexpressed in cases of renal and pulmonary diseases, where the presence of fibrotic tissue degradation is a frequent occurrence. The effectiveness of miR-21 inhibitors in preventing both cardiac fibrosis and neointimal formation has been demonstrated in animal models [18]. While a complete absence of miR-21-5p may not be evident, the recurrent impact of its inhibitors is revealed through the genetic knockout of miR-21 in non-myocyte cells [20]. This emphasizes their vital function in the proper functioning of these cells. A notable increase in the levels of miR-21-5p has been observed in cardiac macrophages and fibroblasts. A recent study on mouse macrophages revealed that the absence of miRNA-21-5p led to resistance to the constriction of the aorta. This was determined by observing structural and functional phenotypes, which were linked to a reduction in inflammatory phenomena. In a subsequent experiment, the administration of antimiR-21 to pigs following an ischemia/reperfusion model resulted in enhanced cardiac function and decreased inflammatory response. Research has indicated that microRNA-21-5p plays a substantial role in promoting fibrosis and inflammation in the myocardium. In light of these findings, a phase II study is currently underway to evaluate the efficacy of LNA-antimiR-21 in the treatment of fibrotic renal disease [19].

The miR29 family consists of four nearly identical variants that are thought to regulate collagen and other matrix proteins. As a result, it has been recognized as a promising target for antifibrotic therapies. A significant study by von Roji et al. [21] demonstrated that the administration of miR-29 mimics led to a decrease in collagen synthesis, thereby enhancing cardiac function. Additionally, the research demonstrated the role of miR-29 mimics in enhancing cardiovascular health.

This concept has been reiterated in various publications, ultimately leading to the development of a final mimic of microRNA-29, designated as MRG-201. MRG-201 has demonstrated efficacy in the treatment of idiopathic pulmonary fibrosis. The fundamental principle underlying this development is the reduction of collagen expression. However, antimiR-29b has been observed to promote the stabilization of the vascular wall. This results in structural changes that lead to the development of abdominal aortic aneurysms in murine models [22,23].

In contrast, Sassi et al. [17] observed that the inhibition of microRNA-29, as opposed to its elevation, prevents cardiac fibrosis. This unanticipated outcome has perplexed the field’s experts. However, the authors have clearly defined the exact nature of the underlying process. Observations of the expression patterns of the miR-29 variants indicate their predominant presence in cardiac myocytes, along with elevated levels detected in cardiac fibroblasts [19,21,22,23].

However, these levels have only been documented in cases where prolonged cultivation has been employed, suggesting a potential temporal element in the regulation of these genes. It has been demonstrated that the principal pathophysiological process by which the induction of fibrosis in fibroblasts occurs through the mechanism of miR-29 activation is through the process of Wnt pathway activation, resulting in the phenomenon of cell hypertrophy and paracrine signaling. Consequently, the inhibition of miR-29 could serve as a viable therapeutic intervention for patients afflicted by myocardial disease. Conversely, the elevation of miR-29 could function as a means of suppressing fibrotic pathways in fibroblasts, thereby addressing skin diseases in patients [19,21,22,23].

Two different investigations [27,30] have assessed the enhanced endothelial downregulation of miR-92a-3p and its disturbance in mouse models of myocardial and vascular lesions. A practical LNA antimiR against miR-92a has been demonstrated to promote angiogenesis (the process by which new blood vessels form) and tissue restoration (the process of regeneration of bodily tissue) in these models [30], which has since been validated in a study based on a porcine ischemia/reperfusion model [27]. The translation of these data was conducted with a focus on clinical application, drawing on the findings of a pharmacological study on antimiR-92a (MRG-110). This cohort included healthy subjects who were administered a single intravenous dose of a drug under investigation. The group under study consisted of healthy subjects who were given a single dose of medication intravenously [133]. It is important to note that, based on the effectiveness of antimiR-92a following the intradermal inoculation route, as demonstrated in preclinical studies using animal models of skin injury, a subsequent phase I clinical investigation was designed using this particular administration method (accessible via ClinicalTrials.gov with the NCT03603431 identifier).

Upregulated expression of miRNA-155-5p has been documented in patient populations afflicted with cardiac inflammatory diseases or in corresponding animal models [29,32]. Bone marrow transplantation experiments in murine models suggest a correlation between the proinflammatory activity of miR-155 and the expression level of NF-κB in macrophages [91], suggesting a potential mechanism through which the inflammatory response can be regulated by this miRNA. On the other hand, an increase in the expression of miR-146a-3p has been observed to counteract this effect [92]. While research has indicated that the inhibition of miR-155 can improve cardiac inflammation in murine models, further investigation is necessary to substantiate the initial findings that macrophage-specific deletion of miR-155 can impede arteriogenesis after vascular injury [28]. Cobomarsen, an antimiR against miR-155, advanced to a phase I study in cutaneous T-cell lymphoma (ClinicalTrials.gov NCT02580552), see Table 2. However, a phase II study was halted due to speculation in cardiovascular studies. While the impact of antimiRNA drugs in other areas has been less than expected, the effects on the heart are minimal.

Despite the discontinuation of several therapeutic developments targeting miRNAs, their impact on the cardiovascular field appears smaller than expected. The data from these drugs can contribute to the development of future heart therapies. For instance, the miRNAs such as miR-17-5p, miR-21-5p, miR-29b-3p, and miR-92a-3p have been the focus of extensive research, and CDR132L is currently under development for the treatment of heart failure. CDR132L is currently undergoing phase II trials and has demonstrated significant potential. The preclinical phase of MiR-132-3p has been completed successfully, with the drug now undergoing clinical trials. These trials are showing rapid progress in terms of efficacy, safety, and benefits. It is noteworthy that research findings have indicated that the genetic deficiency of the miR-132/-212 cluster or the utilization of an antagomir against miR-132-3p can impede the process of pathological cardiac remodeling induced by transverse aortic constriction (TAC) [31]. These observations have led to a review of the potential for miR-132-3p inhibition to be a therapeutic strategy for mouse models of heart failure. In these models, the induction of blood pressure overload is used to create conditions similar to clinical ones [28,31]. In a separate investigative study that used a porcine heart failure model, the researchers demonstrated that microRNA 132 had a prolonged presence in cardiac tissue (with a t1/2 of 3 weeks), accompanied by an advantageous safety profile.

In addition, the authors validated the derepression of predicted microRNA-132 targets. In models of myocardial infarction in pigs, the administration of antimiR-132 resulted in enhanced cardiac function following injury. A similar effect was observed in a separate pig model, wherein the same benefits were seen in the prevention of chronic pressure overload. The initial human study on dose-escalation, conducted as part of phase Ib, focused on patients suffering from heart failure. This study revealed a favorable tolerability profile while providing initial indications for therapeutic efficacy [134,135].

#### 3.3.2. MiRNAs and Coronary Artery Disease

Studies have demonstrated the involvement of miRNAs in the development of atherosclerotic plaque, with their expression levels exhibiting changes in accordance with the progression of coronary artery disease (CAD). According to the findings of certain studies, while microRNAs (miRNAs) may not surpass troponins in terms of effectiveness for diagnosing acute myocardial infarction (AMI), they could contribute to earlier diagnosis and prognosis. Further research is needed to determine the most effective methods for addressing this issue. Reports have documented conflicting findings in patients experiencing stable angina and unstable angina, highlighting the need for more research into miRNA use for diagnosing acute coronary syndrome (ACS).

A document from the German Center for Cardiovascular Research confirmed the advantages of using biomarkers to swiftly identify stable (SA) and unstable angina (UA) [136]. The study yielded significant yet inconclusive findings from three phases of research and the analysis of a substantial number of patients. Initially, 667 miRNAs were identified after screening three patient groups: 10 with ACS, 10 with non-cardiac chest pain (NCCP), and 20 healthy controls. Twenty-five miRNAs were found to be associated with UA (*p* < 0.05). Three miRNAs (miR-1, miR-208a, and miR-208b) were checked in an additional patient group (49 UA patients and 48 NCCP patients). Eight microRNAs (miRNA-19a, miRNA-19b, miRNA-132, miRNA-140-3p, miRNA-142-5p, miRNA-150, miRNA-186, and miRNA-210) were deemed crucial as they are significantly present in patients with UA in the early stages. The third phase validated the diagnostic capability of miRNAs in UA subjects, with 46 UA and 63 NCCP patients from the first cohort analyzed. The respective areas under the curve (AUC) were calculated comparing UA and NCCP patients at baseline. MiR-186 (AUC = 0.78) was the most significant, with miR-132 (AUC = 0.91) and miR-150 (AUC = 0.91) the second- and third-most significant [136].

Researchers assessed circulating miRNAs in patients with CAD and healthy controls to identify biomarkers that could distinguish between SA and UA. The study screened 367 miRNAs and recorded data for three that were more expressed in CAD patients. An increase in miR-NA-337-5p was observed in SA but not in UA. Both SA and UA had increased levels of miR-433 and -NA-485-3p compared to healthy controls, but lacked specificity to SA and UA. An increase in miR-NA-337-5p was observed in SA but not in UA. Both SA and UA had increased levels of miR-433 and -NA-485-3p compared to healthy controls, but lacked specificity to SA and UA. Fourteen previously studied miRNAs were investigated, seven of which were dysregulated. Data indicated significant increases in miR-1, -122, -126, -133a, -139a, and -199a in SA or UA. miR-145 was increased in patients with UA. The statistical analysis supported the predictive value in both groups. Three miRNAs exhibited a predictive value in the SA patient cohort exceeding 0.85: 0.918 for miR-1, 0.929 for miR-126 and 0.851 for miR-485-3p, and 0.92 for miR-1, 0.867 for miR-126 and 0.906 for miR-133a in the UA cohort. No statistical advantage in the AUC area was identified when the two SA/UA arms were compared [137]. The combination of three effective miRNAs distinguished SA and UA patients from non-pathological controls with 90.2% and 87.2% efficiency, but not the two groups with CAD [137]. This study differed from Fichtlscherer et al. [138] in that it assessed the AUC area by correlating various clinical CAD conditions and found that levels of certain miRNAs differed in SA and healthy controls.

To underscore the mounting significance of microRNAs, the most effective approach is to identify their up- or downregulation. In a study of coronary artery disease patients, the diagnostic potential of altered expressed microRNAs was assessed [34]. MicroRNAs that were significantly deregulated in the UA group were used to screen 13 UA patients and 13 patients with non-atherosclerotic chest pain. The categorization of these microRNAs as clusters or members of specific families revealed that some originated from nearby gene loci. Examples include the miR-106b/25/93 and miR-17/19b/20a/92a clusters [138]. However, the miR-21 family 590-5p was classified under the same family as other members due to the presence of a shared 5′ seed sequence. A select group of seven high-level circulating microRNAs were chosen for further study because of their membership in a particular cluster or group. This included the following: miR-106b, miR-25, miR-92, miR-21, miR-590-5p, miR-126*, and miR-451. The study’s sample size included 125 patients, with 45 experiencing unstable UA, 31 with SA, and 37 serving as controls [139].

In light of the mounting importance of microRNAs, it is essential to understand their regulatory mechanisms. Fichtlscherer et al. [138] and Ren et al. [139] initially proposed a method to assess the dosage of miRNAs separately, despite having differing views on the ACS clinical scenario. In patients with CAD due to SA, expression levels of certain miRNAs were found to be lower [140]. In patients with CAD due to UA, increased levels were observed. All seven of these miRNAs were significantly increased in patients with UA compared to those with SA or healthy control subjects, even after adjusting for risk factors. The cluster of miRNAs (miRNA-106b/25, -17/92a, -21/590-5p, -126*, and -451) may be a potential biomarker for unstable angina. Su et al. [141] recently reported on the use of circulating microRNAs as biomarkers. The direct S-Poly(T)Plus method was used to study 203 patients with CAD and 144 age-matched controls. In validation set 2, six miRNAs achieved a sensitivity of 92.8% and a specificity of 89.5% (AUC 0.971, 95% CI 0.948–0.993, *p* < 0.001), validating the direct S-Poly(T) Plus methodology for diagnosing CAD. Plasma fractionation showed that only small quantities of miRNAs accumulated in extracellular vesicles [141].

#### 3.3.3. MicroRNAs and Acute Coronary Syndrome

Supporters of the hypothesis that miRNAs 208b, 499, 133a, 21 and 146a play a significant part in detecting ACS have provided solid support through sophisticated statistical analyses [137,142,143,144,145,146,147,148,149,150,151,152,153]. This assertion has gained formal recognition through two groundbreaking papers that evaluated and recommended specific cardiac and muscle-enriched miRNAs as biomarkers for acute AMI [137,142].

Subsequent to the emergence of evidence in this area, a large-scale study was conducted with the objective of establishing the role of miRNAs in the diagnosis of AMI. In this study, the levels of miRNA-208b and miRNA-499, as well as high-sensitivity troponin (hs-Tn), were measured in a cohort of 510 patients who had experienced an AMI and a control group of 87 individuals who were deemed to be healthy [143]. Davaux et al. [143] found that patients with AMI had higher levels of certain microRNAs compared to healthy individuals. MiRNA-499 showed promise in distinguishing between healthy people and those with AMI, with an area under the curve value of 0.97. However, its diagnostic accuracy compared to measuring high-sensitivity troponin (hs-Tn) alone was not significantly better. If an incorrect AMI diagnosis is made and rectified with additional analysis, the diagnosis does not meaningfully improve.

MiRNAs have lower diagnostic potential than cTnT because of their lower sensitivity and specificity. A study analyzed four different miRNAs (miRNA-1, -133a, -208b, -499) and cTnT in 67 AMI patients and 32 healthy volunteers. All four miRNAs increased within 12 h of onset. On day 14 after the MI, controls had comparable levels. These findings show that these miRNAs may serve as effective biomarkers for AMI [144]. A meta-analysis compared the ability of miRNAs to serve as biomarkers in individuals with and without AMI, combining data from two studies. A total of 192 articles were reviewed, with 19 selected for analysis. Of these, four miRNAs—499, 1, 208b, and 133a—were examined. The AUC values indicated that the biomarkers are reliable. While 133a and 499 are significant for AMI, the role of 208b requires further study [145].

Researchers have studied circulating miRNAs in whole blood, but not yet in subcomponents. Ward et al. [146] used a real-time polymerase-chain reaction system to explore the profiles of different whole blood subcomponents. This included 13 acute myocardial infarction patients with signs of ST-elevation myocardial infarction (STEMI) and non-ST-elevation myocardial infarction (NSTEMI). Arterial blood samples were collected during percutaneous coronary artery intervention (PCI). The protocol requires cell-specific profiling of miRNAs from whole blood, plasma, and platelets. The discussion is based on three conditions: patients with STEMI exhibited higher levels of certain miRNAs, and noted increased expression of specific miRNAs (miR-25-3p, miR-221-3p, and miR-374b-5p), compared to the NSTEMI cohort. The non-coding RNA sequences of the miRNA 30d-5p were found in plasma, platelets and leukocytes in both types of ACS. However, microRNAs 221-3p and 483-5p were only found in plasma and platelets in NSTEMI patients. This suggests differences in the type of cell-specific miRNA between ACS types. The distribution of these microRNAs was also found to vary between patients with STEMI and NSTEMI. The study suggests that patients with myocardial ischemia could be identified by their unique microRNA profiles in their circulating subcomponents [146].

The small number of subjects studied means there is potential bias when validating the diagnostic power of miRNAs in various clinical settings, like ACS, including SA, UA, and AMI, in patients admitted to the emergency department (ED) or undergoing PCI. This condition does not adequately evaluate the relationship between miRNAs and clinical characteristics or their potential prognostic significance. Widera et al. [147] re-examined the diagnostic and prognostic usefulness of cardiomyocyte-enriched miRNAs in various clinical contexts, reigniting speculation about their potential as indicators in ACS. Research examined highly sensitive biomarkers of myonecrosis in 444 ACS patients. Concentrations of several specific miRNAs were assessed via qRT-PCR in plasma samples collected from patients upon admission to the emergency department or those who underwent PCI. A regression analysis established independent connections with levels of the markers hsTnT, miR-1, miR-133a, miR-133b, and miR-208b (all *p* < 0.001). Higher levels of miR-1, miR-133a, and miR-208b were observed in patients who experienced a AMI than those with UA. However, levels of the six investigated non-coding RNA sequences varied widely across subjects with either condition. Levels of miR-133a and miR-208b were significant predictors for mortality risk in both analyses. However, after further adjustment for hsTnT, both miRNAs no longer maintained their independent association with mortality.

Oerlemans et al. [148] conducted a study on the role of circulating miRNAs in ACS. This study built on earlier ones, including comparisons with cardiac injury stability markers. In total, 332 patients with suspected ACS were admitted to a single center for a single evaluation of circulating miRNAs as new biomarkers. The study focused on cardiac miRNAs (miR-1, -208a,-208b, -499) and other miRNAs. Patients with ST-elevation myocardial infarction (STEMI) were not included. Levels of the analyzed miRNAs rose significantly in 106 patients diagnosed with ACS. Levels of miR-NA-208a and miR-146a were higher in non-ST-elevation myocardial infarction (NSTEMI) patients than in those with unstable angina (UA). Levels of miR-NA-499 were lower in UA patients than in NSTEMI patients. Levels of miR-21 were similar in NSTEMI and UA subjects. Furthermore, circulating miRNAs that have been detected in patients suffering from NSTEMI and UA present a significant potential as new early biomarkers with which to assist in the management of individuals suspected of having ACS.

Bai et al. [149] expanded the scope by examining a more varied demographic of individuals with ACS. They used gene chip technology to assess levels of microRNA (miRNA) in patients with SA, NSTEMI, and STEMI. Five individuals from each category and five controls without CAD were included. All participants exhibited three or more risk factors. Microarray analysis was implemented and discrepancies in expression levels were identified and confirmed by qRT-PCR. Patients in the control and SA groups exhibited differentially expressed miRNAs compared to those in the STEMI groups. Substantial increases in miR-941, miR-363-3p, and miR-182-5p were detected in the control, AS, and NSTEMI groups, respectively, compared to the expression levels in patients with STEMI. qRT-PCR analysis revealed increased plasma miR-941 in NSTEMI and STEMI patients compared with those without CAD. MiR-941 expression was significantly increased in the STEMI group compared to SA and NSTEMI. Furthermore, it was higher in patients with ACS and NSTEMI or STEMI, in contrast to patients with SA or without ACS or CAD. These findings suggest that miR-941 is expressed at relatively high levels in patients with ACS and STEMI, potentially indicating its role as a biomarker for ACS or STEMI.

Two studies by Wang et al. [150,151] support novel predictors for AMI and deserve comprehensive discussion. Using microarrays, the authors reported changes in the expression of specific miRNAs in patients with heart disease. However, no significant evidence exists to support this during the initial stage of an AMI [26,28]. The analysis previously investigated the clinical utility of circulating microRNAs in diagnosing and monitoring AMI. A cohort of 17 AMI patients and 28 fit volunteers formed the initial group, and a second group of 9 AMI patients, 9 patients suffering from ischemic stroke, 8 with pulmonary embolism and 12 healthy volunteers formed the subsequent group. Plasma microRNA concentrations and cTnI concentrations were measured in the blood using real-time PCR and ELISA assays, respectively. AMI patients exhibited elevated levels of two circulating miRNAs—miR-21-5p and miR-361-5p—while the concentration of a third, miR-519e-5p, was reduced. These changes were strongly linked to the levels of another marker, cTnI. At an evaluation of the ROC, the three markers had noteworthy diagnostic accuracy for AMI, demonstrating a high AUC value. Combining the three miRNAs significantly increased diagnostic precision. Wang et al. established that these circulating microRNAs can act as new biomarkers for AMI [150]. In their second research, Wang et al. [151] investigated the link between high-risk characteristics in patients with NSTEMI. They used a Global Registry of Acute Coronary Events (GRACE) analysis and extracted RNA from the whole blood of 199 patients with NSTEMI, conducting whole-genome sequencing. To verify the study’s reliability, 13 high-risk clinical traits were examined using generalized linear models. The GRACE risk score has been extensively validated for mortality among patients with NSTEMI. A total of 205 risk factor associations were found to be nominally significant (*p* < 0.05). Upon eliminating the false discovery rate of 5%, it was observed that chronic heart failure had a substantial association with lower levels of circulating miR-3135b, -126-5p, -142-5p, and -144-5p.

Two recent studies [152,153] merit consideration. Kaur et al. [152] assessed dysregulated miRNA biomarkers in CAD by screening 140 studies with sufficient data. Their review collated data on the most common miRNAs in CAD: miR-1, miR-133a, miR-208a/b, and miR-499. These were also found in the heart. Some miRNAs were consistently identified in multiple studies and had different expression levels in the ACS and stable CAD groups. Certain miRNAs have been used as biomarkers in ACS patients with high levels of miR-499, miR-1, miR-133a/b, and miR-208a/b, and in stable CAD patients with higher levels of miR-215, miR-487a, and miR-502. Elevated plasma levels of miR-21, miR-133, and miR-499 are good biomarkers that can differentiate between ACS and stable CAD. Particular attention should be given to miR-499, which has shown a link between its levels and AMI. However, these miRNAs should be interpreted cautiously as the studies were mostly based on a small number of miRNAs.

Zhelankin et al. [153] studied circulating miRNAs as non-invasive biomarkers of cardiovascular disease in CAD and ACS. They highlighted concerns over data inconsistency, likely due to pre-analytical and methodological discrepancies. The research sample included 136 participants with CAD or ACS (including NSTEMI and STEMI patients), and controls (outpatients with CAD or hypertension). Patients with ACS had higher plasma levels of miR-21-5p and miR-146a-5p, and lower levels of miR-17-5p, compared to controls. Within the ACS patient group, no significant differences in plasma levels were found. The findings suggest that elevated plasma concentrations of miR-146a-5p and miR-21-5p are general biomarkers for ACS, while decreased levels of miR-17-5p are a universal biomarker for CAD. Elevated plasma concentrations were found for all three miRNAs in the ACS group, regardless of troponin levels, and between patients with STEMI and NSTEMI. Therefore, elevated plasma levels of miR-146a-5p and miR-21-5p can identify ACS, and decreased levels of miR-17-5p, CAD.

#### 3.3.4. Role of MiRNA in Stent Restenosis

Changes in the levels of miRNAs have been observed in cases of in-stent restenosis, a problem that can arise after PCI. It has been demonstrated that both bare metal stents and drug-eluting stents have the potential to manifest abnormal intra-stent neointimal formation. However, with bare metal stents, the occurrence of vascular lesions promotes uncontrolled neointimal formation and restenosis due to atypical proliferation and smooth muscle cell migration [154,155,156]. While drug-eluting stents have effectively mitigated tumor proliferation, the desired significant reduction in restenosis risk has not been consistently achieved. However, the heightened risk of late thrombosis associated with drug-eluting stents is a concern. The promotion of late thrombosis in these stents is attributed to the ability of endothelial cells to regenerate after vascular wall injury and the prolonged healing process [157,158,159,160,161,162].

The impact of stent type—bare metal vs. drug-eluting—on modulation of microRNA levels and their functionality in porcine, murine, and in vitro models has been examined [163]. In porcine models, miRNA levels were analyzed in coronary arteries. The findings indicated increased proinflammatory miRNAs-21 in stented arteries. In stent-KO mice, there was a decrease in neointimal thickness and area. Subsequent analysis revealed a greater proportion of anti-inflammatory M2 macrophages in the stent-KO mice. The study found that stenting with miR-21 reduced neointimal proliferation due to a shift towards M2 macrophage differentiation, linked to reduced smooth muscle cell proliferation and migration, while preserving endothelial cell function. This indicates that miR-21 may play a role in stimulating inflammation and vascular remodeling following stent implantation. Experiments are underway to test the effects of new medication that regulates the expression and plasma levels of miRNA-21. The objective is to enhance the effectiveness of existing drug-eluting stents used to treat thrombosis [163].

**Figure 5:**
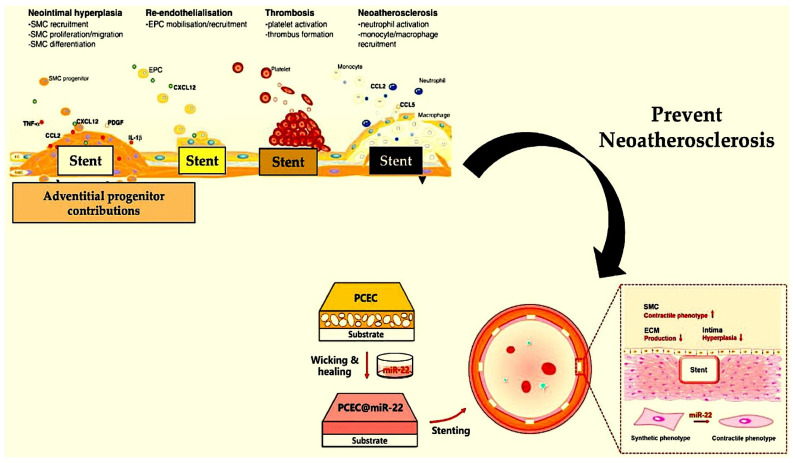
The subsequent illustration details the preparation of the PCEC@miR-22-coated stent. The porous coating of the PCEC was loaded with the miR-22 through a wicking process, followed by encapsulation through self-healing. Ref. [164].

In the humanized model, Wang et al. [162] explored the function of microRNAs in myointimal hyperplasia/in-stent restenosis, revealing that miR-21 levels were elevated in human tissue samples from restenosis patients compared to coronary heart disease patients. In the model, the effects of miR-21 were blocked using an intravenous anti-21 solution. A reduction in the levels of vascular miRNA-21 has been shown to result in a decrease in occlusion and off-target effects. These effects include lowered expression in the liver, heart, lungs, and kidneys, as well as elevated serum creatinine. The use of anti-21-coated stents in local suppression was found to be effective in reducing restenosis compared to the use of bare metal stents. There were no observed off-target effects. These results indicate that anti-21 coated stents could provide an effective therapeutic solution for reducing restenosis. Recently, Wang et al. [162] developed a cardiovascular stent that delivers miRNA-22 to regulate SMCs. The stent is designed using this template molecule, and its function is supported by a self-healing encapsulation process. Furthermore, it was observed that the sustained delivery of miR-22 through stenting significantly enhanced the contractile properties of SMCs without impeding endothelium cell (EC) proliferation. The results showed predominant EC growth, with an EC/SMC ratio of 5.4. PCEC@miR-22-coated stents have been shown to minimize inflammation, prevent excessive changes to the stent’s structure, and inhibit the secretion of extracellular matrix, reducing the risk of in-stent restenosis. This finding establishes a foundation for the development of coating platforms for the delivery of miRNAs to cardiovascular stents. This innovation has the potential to be expanded to other medical devices, see Figure 5.

### 3.4. Oligonucleotide-Based Therapy Findings

Antisense oligonucleotides function by selectively inhibiting miRNA molecules through their complementary binding. Unlike other RNA molecules, such as ribosomal RNA (rRNA), which can integrate into the RISC, ASOs act as single strands, allowing for targeted inhibition without disruption [165]. Significant alterations in the levels of endogenous miRNA, ranging from 3- to 4-fold up to 30-fold deregulation, have a substantial impact on targetomes and disease manifestations. Antisense oligonucleotides inhibit miRNA by acting as single strands, without integrating into the RISC [165]. Changes in levels of endogenous miRNA, from 3- to 4-fold up to 30-fold deregulation, have a dramatic impact on targetomes and disease phenotype [16,166,167,168,169].

It has been demonstrated that certain clusters of miRNAs can exhibit cooperative functionality, a notable example being the miR-106b~25 cluster [168]. Individual miRNAs have the ability to regulate multiple levels of a cellular process. Examples include the miR-378a-3p, the miR-29 family, and miR-365-3p [16,17,170]. The combination of miRNAs has been shown to enhance their capacity to modify diseases. Reverse complementary base pairing is a key technology in the field of therapeutic development, where it is employed to target and inhibit a wide range of oligonucleotides. These include ASOs that cause RNase H cleavage and morpholinos that mask translation initiation or splicing regions. Other examples include siRNAs and miRNA inhibitors [165]. Currently, ten drugs have received approval for use based on ASOs. Several other products are currently undergoing clinical studies. Inclisiran is a first-in-class ASO designed for the treatment of cardiovascular disease. It has been shown to reduce cholesterol and prevent atherosclerosis [171]. As shown in Table 2, the most advanced drug candidates for treating hepatitis C are antimiRs: miravirsen (anti-miR-122/SPC3649) and RG-101 (antimiRNA/miRNA-122). However, these drugs have lost medical need due to the exceptional efficacy of other medications and the development of viral resistance. However, these findings demonstrate the feasibility of microRNA-based therapy. At the start of 2002, 19 microRNA-based therapeutics’ trials had been completed or were underway. Two additional trials involving miR-103/107-3p (RG-125/AZD4076) and another involving miR-155-5p (cobomarsen/MRG 106) were discontinued by sponsors for strategic reasons, see Table 5 [172,173,174,175,176,177,178,179,180,181,182,183,184,185,186,187,188,189,190,191,192,193,194,195,196,197].

Second-generation modified molecules have been eliminated. These are used in RNA-based therapies to reduce the immune response to RNA drugs. Research has shown that 2′-ribose modifications on siRNAs can effectively halt TLR stimulation, particularly when utilized on uridines in GU-rich sequences [172,173,174]. Initial findings regarding the efficacy and safety of the miR-34a mimic MRX34 have been encouraging. MRX34 has demonstrated robust anti-tumor activity in ongoing studies. However, the MRX34 trial was halted due to immune-related side effects [175,176]. The side events in question have been linked to inflammation, cytokines, enterocolitis, hepatic failure, hypoxia, and respiratory failure [72].

It was discovered that the immune effect of miR-34a was unexpectedly strong. Preclinical studies in mice revealed no signs of immunogenicity [177]. Another study failed to demonstrate immune stimulation when the same vehicle was used to deliver ssDNA molecules (PNT2258) [178]. This indicates that the delivery vehicle is not the root cause of the issue [178]. MRX34 is present in the highest concentrations in the liver, bone marrow, and spleen of non-human primates [179]. A decrease in the number of miR-34a target genes in white blood cells was observed in the pharmacodynamic analysis of patients in the phase I study. Additionally, there was an increase in miR-34a levels in tumor tissue. The treatment protocols for the three patients who responded to MRX34 therapy (which had a 4% response rate) are not specified. It is important to note that miR-34a targets the immunotherapy target PD-L1, which may have led to the observed responses. Currently, there are no microRNA mimics or overexpressions for cardiovascular indications in the clinical trial stage.

#### 3.4.1. Clinical Trial Development of MiRNA-Based Cardiovascular Therapeutics

Recent findings suggest that the impact on the field of cardiovascular science has been less significant than expected, although a number of RNA interference-based therapeutic interventions targeting other medical conditions have been discontinued (for instance, miravirsen, RG-101, cobomarsen, and AZD4076). Conversely, preclinical and clinical evidence furnish invaluable insights that facilitate the conception and evaluation of efficacious cardiovascular therapies that leverage these molecules, including those that have been discontinued. These molecules have undergone extensive investigation in both laboratory and clinical contexts. As illustrated in Table 3, the development of the miR-132-3p inhibitor (CDR132L) holds particular significance for the treatment of heart failure. This inhibitor has the potential to be the first drug in the category of RNAi-based therapeutics to be employed for the treatment of cardiovascular conditions. It is currently undergoing testing as part of phase II.

With the exception of ASOs, there are no near clinical miRNA mimics or overexpressions currently being used in cardiovascular indications. It is imperative to acknowledge the criticality of the temporal and dosage parameters of microRNA-boosting therapy, as substantiated by the complications observed in the MRX-34 antitumor trial [78] and the deleterious impact of both prolonged miR-199a and miR-92a expression in mouse models. As demonstrated in the research, the timing and dosage of microRNA-boosting therapy are pivotal. This is supported by the findings of the MRX-34 antitumor trial [78] and the deleterious impact of both prolonged miR-199a and miR-92a expression in mouse models [193,195].

#### 3.4.2. Looking at How Oligonucleotides Move: Questions to Ask and Big Challenges to Overcome

**Figure 6:**
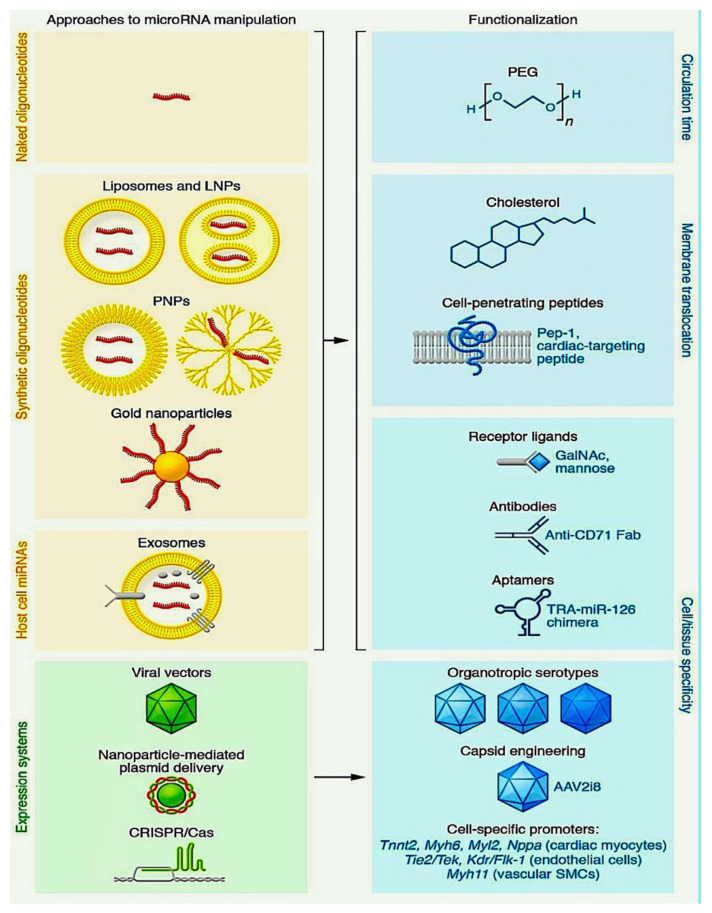
The figure illustrates molecular vehicles for regulating microRNAs and demonstrates the functionalization process. Modified nucleotides in synthetic oligonucleotides have been shown to improve nuclease resistance, allowing them to be used as “naked” molecules. The incorporation of these elements into lipids has been shown to enhance cellular uptake through the process of endocytosis. Additionally, metallic particles, including gold, can be used to deliver oligonucleotides and plasmids. Exosomes carrying microRNA can be obtained from natural sources or designed for better loading or targeting [196,197]. Oligonucleotides, or their delivery vehicles, can be modified to improve circulation time, membrane penetrance, or cell- or tissue-specific delivery. The transferrin receptor aptamer (TRA) is an example of such a mechanism. Viral vectors are utilized to express or genetically inactivate microRNAs or their targets. AAV engineering has been demonstrated to enhance transduction and/or tropism, and the use of cell type-specific promoters has been shown to further optimize the process [198,199]. Tnnt2 (cardiac troponin T2), Myh6 (myosin heavy chain 6), MyL2 (myosin light chain 2), and Nppa (natriuretic peptide A) are promoters used in cardiac myocytes. Tie2/Tek and Kdr/Flk-1 (kinase insert domain receptor/fetal liver kinase 1) are used in endothelial cells. Myh11 (myosin heavy chain 11) is used as a promoter in vascular smooth muscle cells [100,199,200,201,202,203,204,205,206,207].

Achieving efficient oligonucleotide concentrations in target tissues or cells is challenging. Figure 6 summarizes several strategies, many of which hold hope for cardiovascular applications.

However, oligonucleotides have hydrophilic properties and do not readily cross membranes [110]. Additionally, their distribution into cardiovascular tissue may be overridden by renal filtration, and the presence of monocytes in the liver, spleen and bone marrow decreases cardiovascular accessibility [197]. In the myocardium, this results in low cellular incorporation, although this phenomenon [198,199,200,201] is enhanced in pathological conditions [202]. Oligonucleotides face the problem of being sequestered in endosomes after endocytosis [194,195]. LNA antimiRs can overcome these hurdles by penetrating membranes. Many cardiovascular studies are conducted without formulation of the antimiR, as shown in Table 6 [203,204,205,206,207,208,209,210,211,212,213].

Strategies to improve oligonucleotide circulation, transport, delivery and tropism include lipid, polymer, hybrid and metal-based nanoparticles [165]. Oligonucleotides are also conjugated with polyethylene glycol (PEG) to delay clearance. Cholesterol can be attached to oligonucleotides to help them cross the membrane, and to nanoparticles. Cell-penetrating peptides (CPPs) have demonstrated cardiovascular efficacy in vivo [200,201]. A CPP conjugate of eteplirsen is currently being investigated in a Duchenne muscular dystrophy phase II clinical trial (ClinicalTrials.gov NCT04004065).

By coupling oligonucleotides or miRNA vehicles to receptor ligands or other cell-targeting molecules, the most efficacious cellular tropism is anticipated [214,215,216] (Figure 6). To serve as binding sites for oligonucleotides, the molecules must be able to attach to cell surface proteins and must not hinder the translocation or action of the medication or cause unwanted side effects. This strategy uses a therapeutic approach using an siRNA linked to a CD71 Fab’ fragment to target the heart and skeletal muscle in mice. This has been shown to be effective in treating muscular dystrophy [214]. Centyrins, fibronectin-3 derivatives, can be designed for specificity and affinity and coupled to oligonucleotides [215]. A folate-coupled antimiR-34-3p in mice affects tumors preferentially [216]. Oligonucleotides linked to GalNAc, a natural ligand of asialoglycoprotein receptor 1, which is highly expressed in liver cells, are clinically advanced, making them ideal for liver-targeted therapy [2]. Other sugars may also be useful for cell-specific oligonucleotide release, such as mannose, whose receptor is mainly expressed on macrophages. Aptamers have also been evaluated in conjunction with siRNAs [217]. One aptamer enhances miR-126-3p release by binding to the transferrin receptor [218].

Adeno-associated viruses (AAVs) are used to transport genetic information. Their organotropic serotypes can be optimized through capsid engineering. One example is AAV2i8, a chimera of an AAV2 inner loop mutant and AAV8 [200,201,219,220], which is particularly effective in transducing myocytes [221]. This construct has been used to drive inhibitor-1 expression in a porcine model of cardiac ischaemia [222]. It is currently being evaluated in a phase I clinical trial (ClinicalTrials.gov NCT04179643). Targeted evolution has produced AAVs with superior muscle cell specificity and transduction efficiency [223]. The use of specific promoters for gene regulation in different cardiovascular cell lines broadens possibilities. In addition to the benefits provided by viral vectors, molecular genetic tools like CRISPR/Cas plasmids can be introduced non-virally. The delivery of plasmids for non-coding RNA will determine whether it is effective for microRNA expression [222], see Figure 6.

### 3.5. Clinical Applications and Areas of Uncertainty

Globally, cardiovascular disease (CVD) is a significant health concern, impacting mortality and morbidity on a broad scale, including in the Middle East [202,224,225,226,227]. Annually, CVD accounts for an approximated 17.9 million deaths worldwide [228]. It is associated with various conditions, such as heart disease, heart attacks, strokes, and ischemic heart disease [229,230]. It is particularly concerning that heart disease and stroke account for over 80% of all CVD-related deaths, with a worrying one-third of these occurring in patients younger than 70 years of age [231]. In recent years, there has been a significant focus on developing innovative therapeutic solutions for various diseases. These include monoclonal antibodies that target proprotein convertase subtilisin/kexin type 9 (PCSK9) and strategies designed to reduce low-density lipoprotein cholesterol (LDL) levels. These approaches have shown promising results in addressing these health concerns with effectiveness [226,227,232]. It is an established fact that lowering LDL-C levels brings many benefits. These include a decrease in the risk of heart attacks, enhanced vessel wall stability, reduced vascular wall inflammation and the consequent strengthening of atherosclerotic plaques [233].

PCSK9 is an essential regulatory protein that controls cholesterol levels by binding to LDL receptors on the surface of hepatocytes [234,235,236]. It is well-established that LDL receptors have a significant function in the uptake of LDL-C from the circulation. However, research has shown that PCSK9 binds to LDL receptors, which in turn causes their degradation. The result of this process is that fewer LDL receptors are available for use, thus leading to a decrease in the amount of LDL that is cleared from the circulation [234,237]. Accordingly, an accumulation of LDL in the blood has been shown to significantly increase the risk of CVD [238]. Therefore, PCSK9 is regarded as a potential target for reducing LDL-C [239]. A novel strategy for targeting PCSK9 using RNA-based therapies has emerged over the past few [240,241]. In the context of this scenario, inclisiran, a first-in-class small interfering RNA (siRNA) developed to specifically degrade messenger RNA (mRNA) responsible for PCSK9, has demonstrated significant potential in addressing high cholesterol in patients with a high risk of cardiovascular events [242]. Research indicates that reduced PCSK9 levels allow LDL receptors to remain active on the surfaces of hepatocytes. This, in turn, promotes the elimination of LDL-C from the circulatory system [243].

Inclisiran provides substantial practical benefits, largely attributable to its innovative biannual dosing schedule. Administered by healthcare professionals under the skin twice a year following an initial dose to get things started, inclisiran reduces the daily or biweekly treatment burden typically associated with the pharmaceutical product [244]. Adherence rates are significantly higher when patients receive their therapy during a routine medical visit than when they self-administer products such as PCSK9 monoclonal antibodies [245], and the current dosing regimen has been developed to reflect this. The SPIRIT trial demonstrated that the incorporation of inclisiran into primary care services enhances patient compliance and streamlines treatment processes for both patients and healthcare professionals [246]. Furthermore, the targeted action of inclisiran limits systemic uptake, thereby enhancing its profile in terms of safety [247]. Extended trials, including ORION-3 and ORION-8, have substantiated this favorable safety profile, demonstrating no substantial escalation in significant adverse events [244]. In light of the aforementioned characteristics, inclisiran is deemed to be especially useful within high-risk populations, including those who face difficulties with respect to adherence, or for whom access to healthcare services is constrained [247].

Inclisiran’s unique characteristics make it particularly well-suited for use in high-risk populations, such as those who may encounter challenges in adhering to the treatment regimen or have limited access to healthcare services [247]. However, the long-term impact may lead to ongoing adverse reactions that are challenging to reverse, as the drug’s activity persists without early intervention. This characteristic necessitates careful consideration when administering inclisiran, especially to patients who may be more susceptible to adverse event [248].

As outlined in a previous study by Yang et al. [249], cardiac conduction regulatory RNA (CCRR) has been identified as an antiarrhythmic lncRNA. A recent study was conducted to evaluate the effects of CCRR on SERCA2a and the associated Ca^2+^ homeostasis in MI. The results of the study demonstrated that the expression of CCRR via AAV9-mediated delivery successfully reversed ischemia-induced contractile dysfunction to a certain extent. In addition, the expression of CCRR alleviated abnormal Ca^2+^ homeostasis and reduced the heightened methylation level of SERCA2a following MI. These effects were also observed in CCRR-overexpressing transgenic mice. A conserved sequence domain of CCRR mimicked the protective function observed with the full-length version. Furthermore, silencing CCRR in healthy mice led to intracellular Ca^2+^ overloading of cardiomyocytes. CCRR has been shown to increase SERCA2a protein instability by upregulating FTO expression. The direct interaction between CCRR and FTO protein was characterized by RNA-binding protein immunoprecipitation (RIP) analysis and RNA pulldown experiments. Research has identified activation of NFATc3 as an upstream mechanism responsible for CCRR downregulation in MI. This study shows that CCRR is a protective long non-coding RNA (lncRNA). It does this by maintaining the function of FTO. This, in turn, reduces the m6A RNA methylation level of SERCA2a. This ultimately preserves calcium homeostasis for myocardial contractile function in MI. Consequently, CCRR can be regarded as a potential therapeutic approach with a beneficial role in cardiac pathology [250].

The preclinical studies discussed above indicate that all cardiovascular cell types can be targeted by RNA inhibitor drugs, and that inhibiting these molecules can have significant effects on cardiovascular function. This suggests a strong potential for further exploration of these drugs as novel candidates for treatment. Nevertheless, there are still many challenges and questions to be addressed on the way towards developing RNA-based therapeutic solutions in general. In most of the animal studies to date, the phenotypic effects of RNA interference have only been studied in the target tissue of interest, which might overlook off-target effects in additional tissues. Furthermore, the doses utilized in the majority of studies are not likely to be therapeutically viable. Subsequent preclinical studies will be instrumental in determining the most suitable dosing regimens, with the objective of identifying the lowest effective doses while aiming to mitigate undesirable side effects.

## 4. Future Direction

Although there is a lot of potential for the use of inclisiran in the treatment of certain conditions, there are some limitations to the current body of evidence supporting this use. The absence of direct cardiovascular outcome data necessitates reliance on surrogate endpoints, such as LDL-C reduction. Real-world studies are essential for validating the product’s broad applicability by assessing its long-term safety, adherence, and cost-effectiveness across diverse patient populations. Furthermore, exploring combination therapy with other lipid-lowering agents could optimize cardiovascular outcomes. Future research should also prioritize expanding the evidence base among underserved populations, including those in low-resource settings, to ensure that the benefits of inclisiran are widely accessible. These efforts will refine inclisiran’s clinical utility and solidify its role in the global management of cardiovascular disease [251].

## 5. Conclusions

The advances made in the field over the past decade are exemplified by the increasing number of clinical trials targeting microRNAs, which has culminated in the first clinical trial of an anti-mRNA in cardiovascular therapeutics. The presence of unidentified microRNAs indicates a wider spectrum of potential disease targets and applications for microRNA therapeutics than is currently apparent. However, as highlighted in a comprehensive review of the extensive descriptive literature on microRNAs, the field is confronted with the challenge of rigorously confirming the functions of microRNA candidates [249,250]. It is therefore imperative that therapeutic design is improved and the risk of attrition is decreased by combining microRNA manipulation in disease models, omics technologies and thorough preclinical evaluation. Despite the advent of synthetic oligonucleotides that have surmounted substantial challenges, concerns persist over the delivery and administration of such molecules. This is particularly salient in the context of cardiovascular tissue. The process of incorporating oligonucleotides into cardiovascular tissue is not efficient. Following the enhancement of the pharmacokinetics of oligonucleotides, the necessity for specific delivery methodologies, such as local catheter-based administration, will be negated. Furthermore, the potential exists for the tailoring of oligonucleotides to enhance not only cellular absorption but also cellular targeting. The present state of this field is underdeveloped, and significant investment will be required to develop methods for the screening of ligands and chemical coupling to oligonucleotides, as well as assays for the measurement of cellular oligonucleotide concentrations.

Significant advancements have been made in the diagnosis and treatment of cardiovascular diseases over the last decade. However, further elucidation is necessary to fully comprehend the findings related to numerous miRNAs. A more extensive range of disease states and therapeutic applications is poised to emerge based on the findings from this investigation. It will be necessary to conduct further investigations into miRNAs in order to gain a better understanding of their role in the field of cardiology [223]. This will enhance the development of new therapies and prevent attrition. The strategic modulation of miRNAs in CVD models, in conjunction with omics technologies and extensive trials, has yielded positive results. In an exploratory analysis of 3655 patients (ORION-9, ORION-10, and ORION-11), the addition of inclisiran was linked with a 26% decreased probability of major adverse cardiovascular events and a lower risk of fatal and nonfatal myocardial infarction compared to placebo. It should be noted that the aforementioned trials were not conceived to directly evaluate major adverse cardiovascular events as the primary endpoint [252].

Overcoming hurdles to deliver these molecules remains an issue. This is particularly relevant to cardiovascular tissues, which poorly absorb oligonucleotides. We must specifically modify oligonucleotides to improve their absorption and specificity. This area of pharmaceutical research needs more attention, with uncertainty surrounding the optimal concentrations of cellular oligonucleotides. Significant efforts will be necessary to screen and chemically link ligands to oligonucleotides.

Implementing miRNAs is experimental, but it has potential in diagnosing, prognosing and treating cardiovascular diseases. It might also benefit the next generation of stents. Its application, whether alone or in combination with existing biomarkers, may become standard in the near future, especially when diagnostic uncertainty exists. Further studies are required to confirm its applicability in typical clinical settings. 

## Figures and Tables

**Table 1:** Narrative review searching strategies [62].

Items	Specification
Date of Search (specified to date, month and year)	From January 2026 to Avril 2026
Databases and other sources searched	MEDLINE, Embase, and the Cochrane Library
Search terms used (including MeSH and free text search terms and filters)	MiRNA, microRNAs, long non-coding RNAs, non-coding RNAs, ncRNA therapeutics, microRNA biomarkers and microRNA functions. ‘RNA therapeutics’, ‘Medicines Agency’, ‘Acute coronary syndrome’ and ‘Coronary artery disease’.
Timeframe	Up to Avril 2026
Inclusion and exclusion criteria (study type, language restrictions, etc.)	English language; inclusion criteria: the manuscripts under consideration here all concern research and clinical studies on miRNAs.
Selection process	One author independently selected articles after screening for duplicates.

**Table 2:** Studies on RNA-based therapies approved by the FDA and/or the European Medicines Agency.

Study Type/Acronym NCT Number	Type RNA/Treatment	Participants (N)/Mode of Delivery	Primary Outcome Measures	Disease	Target Gene and Route	FDA and/or EMA Approval Year/Study Status
Multicentric Non-RCT/ILLUMINATE-C NCT04152200 (EU Trial/CTIS) Number) 2023-503382-29-00	21 nt ds-siRNA Lumasiran (Oxlumo, ALN-GO1)	N (21) Cohort A SC Cohort B SC	Cohort A: Percent Change in Plasma Oxalate From Baseline to Month 6 Cohort B: Percent Change in Pre-dialysis Plasma Oxalate From Baseline to Month 6	Primary hyperoxaluria type 1	Hydroxyacid oxidase 1 (*HAO1*) mRNA	2020 (EMA), 2020 (FDA) Phase III
Multicenter RCT ORION NCT03397121	21 nt ds-siRNA Inclisiran (Leqvio, ALN-PCSsc)	N (482) Experimental Inclisiran SC Sodium 300 mg injection on Day 1, Day 90 then every 6 months. Placebo Comparator Saline Solution SC Placebo injections of saline solution on Day 1, Day 90 then every 6 months.	Percentage Change in LDL-C From Baseline to Day 510 Time-adjusted Percentage Change in LDL-C Levels From Baseline After Day 90 and up to Day 540	Atherosclerotic CVD, elevated cholesterol, homozygous/heterozygous familial hypercholesterolaemia	Proprotein convertase subtilisin/kexin type 9 (*PCSK9*) mRNA	2020 (EMA) 2021 (FDA) Phase III
Multicenter RCT/COMPASS NCT02300233	20-mer ASO Volanesorsen (Waylivra)	N (114) Placebo Comparator SC Volanesorsen-matching placebo administered once-weekly for 26 weeks. Experimental Volanesorsen SC Volanesorsen 300 mg administered once-weekly for 26 weeks. Experimental Volanesorsen SC Volanesorsen 300 mg administered once-weekly for 13 weeks, then biweekly for 13 weeks.	Percent Change in Fasting Triglycerides (TG) From Baseline to Month 3	Familial chylomicronaemia syndrome	Apolipoprotein CIII (*APOC3*) mRNA	2019 (EMA) Phase III
Multicenter RCT NCT04060199	21-mer ASO Viltolarsen (Viltepso, NS-065, NCNP-01)	N (77) Experimental Viltolarsen IV (deletion Exon 53) 80 mg/kg infusion for up to 48 weeks. Placebo Comparator IV (deletion Exon 53) placebo infusionsfor up to 48 weeks.	Change From Baseline in Time to Stand (TTSTAND) Velocity	Duchenne muscular dystrophy	*DMD* pre-mRNA splicing (exon 53 skipping)	2020 (FDA) Phase III
Multicenter RCT NCT02452372	21 nt ds-siRNA Givosiran (Givlaari)	N (40) Active Comparator SC Givosiran (ALN-AS1) Placebo Comparator SC Sterile Normal Saline (0.9% NaCl)	Givosiran’s safety is evaluated by the proportion of subjects experiencing AEs, SAEs and AEs leading to drug discontinuation.	Acute hepatic porphyria	Delta aminolevulinic acid synthase 1 (*ALAS1*) mRNA	2020 (EMA), 2019 (FDA) Phase I
Multicenter RCT ENVISION NCT03338816	21 nt ds-siRNA Givosiran (Givlaari)	N (94) Experimental Givosiran SC 2.5 mg/kg administered (SC), monthly (QM), for 6 months during the 6-Month Double-blind (DB) Period, followed by givosiran 2.5 mg/kg or 1.25 mg/kg SC, QM for 29 months during the Open-label Extension (OLE) Period. Placebo/Givosiran SC Matching placebo (normal saline [0.9% NaCl]) was administered SC, QM, for 6 months during the 6-Month DB Period, followed by givosiran 2.5 mg/kg or 1.25 mg/kg SC, QM for 29 months during the OLE period.	Annual rate of porphyria attacks in acute hepatic porphyria participants.	Acute hepatic porphyria	Delta aminolevulinic acid synthase 1 (*ALAS1*) mRNA	2020 (EMA) 2019 (FDA) Phase III
RCT/ESSENCE Multicenter NCT02500381	25-mer ASO Casimersen (Amondys 45, SRP-4045) Golodirsen (Vyondys 53, SRP-4053)	N (222) Experimental SRP-4045 IV (deletion Exon 45) SRP-4045 at 30 mg/kg for up to 96 weeks in the double-blind. Open-label extension period SRP-4045 at 30 mg/kg/week IV infusions for 48 weeks (up to Week 144 in the study Experimental SRP-4053 IV (deletion Exon 53) SRP-4053 at 30 mg/kg for up to 96 weeks in the double-blind. Open-label extension period SRP-4053 at 30 mg/kg/week IV infusions for 48 weeks (up to Week 144 in the study). Placebo Comparator IV Placebo followed by SRP-4045 or SRP-4053 SRP-4045 or SRP-4053 placebo-matching infusions, weekly, at 30 mg/kg for up to 96 weeks in the double-blinded period. Open-label extension period SRP-4045 or SRP-4053 at 30 mg/kg/week infusions for 48 weeks (up to Week 144 in the study).	Change from baseline in 4-Step Ascend velocity at week 96.	Duchenne muscular dystrophy	*DMD* pre-mRNA splicing (exon 53 skipping)	2019 (FDA)
Non-RCT Multicenter NCT02510261 NCT01961921 (study 003) NCT01960348 (study 004)	21 nt ds-siRNA Patisiran (Onpattro)	N (211) Experimental: Prior Placebo IV Group of Study 004 Placebo and completed parent study ALN-TTR02-004 (NCT01960348) 0.3 milligrams per kilogram (mg/kg) patisiran once every 3 weeks (Q3W) up to 65.5 months. Experimental: Prior Patisiran IV Group of Study 004 Patisiran and completed parent study ALN-TTR02-004 (NCT01960348) 0.3 mg/kg patisiran IV Q3W up to 66.9 months. Experimental: Prior Patisiran IV Group of Study 003 Patisiran and completed parent study ALN-TTR02-003 (NCT01961921) were enrolled to receive 0.3 mg/kg patisiran IV Q3W up to 61.4 months.	Percentage of Participants With AEs Leading to Study Discontinuation	Hereditary transthyretin amyloidosis	Transthyretin (*TTR*) mRNA	2018 (EMA), 2019 (FDA) Phase III
RCT Multicenter NCT01737398	20-mer ASO Inotersen (Tegsedi, AKCEA-TTR-LRx)	N (173) Active Comparator: Inotersen SC 300 mg 3 times on alternate days in the first week and then once-weekly for 64 weeks Active Comparator: Placebo SC 3 times on alternate days in the first week and then once-weekly for 64 weeks	mNIS composite score changed by + 7 at week 66. Change from baseline in the Norfolk QoL-DN questionnaire at week 66.	Hereditary transthyretin amyloidosis	Transthyretin (*TTR*) mRNA	2018 (EMA), 2018 (FDA) Phase II/III
Non-RCT Multicenter USA NCT02255552	30-mer ASO Eteplirsen (Exondys 51)	N (110) Experimental: Treated Group eteplirsen IV (deletion Exon 51) 80 patients 30 mg/kg weekly for 96 weeks, followed by a safety extension (not to exceed 48 weeks). No Intervention: Untreated Group placebo IV (no deletion Exon 51) 30 patients not will not receive eteplirsen.	Change From Baseline in the 6MWT Distance at Week 96	Duchenne muscular dystrophy	Dystrophin (*DMD*) pre-mRNA splicing (exon 51 skipping)	2016 (FDA) Phase III
RCT EMBRACE Multicenter NCT02462759	18-mer ASO Nusinersen (Spinraza, ASO-10-27)	N (21) Experimental: Nusinersen intrathecal injection. Sham Comparator: Sham Procedure Small needle prick on the lower back at the location where the IT injection is normally made.	Adverse AEs and SAEs Change From Baseline in ECGs, vital signs, head circumference, chest circumference, arm circumference, weight for age, weight, head to chest circumference ratio, body length, in neurological examination autcomes, aPTT, PTT, INR. Presence of Urine Total Protein Post-baseline	Spinal muscular atrophy	Survival of motor neuron 2 (*SMN2*) pre-mRNA splicing (exon 7 inclusion)	2017 (EMA), 2016 (FDA) Phase II
RCT Multicenter USA NCT00770146	20-mer ASO Mipomersen (ISIS 301012, Kynamro)	N (158) Experimental: Mipomersen SC 200 mg once a week for 26 weeks. Placebo Comparator: Placebo SC Injection 9 mg of sodium chloride, 0.004 mg of riboflavin, filled to 1 mL with water once a week for 26 weeks.	Percent change from baseline in LDL-C at primary efficacy time point. LDL-C at baseline and primary efficacy time point.	Homozygous familial hypercholesterolaemia	Apolipoprotein B mRNA	2012 (EMA), 2013 (FDA) Phase III
RCT Multicenter USA NCT00002187	21-mer ASO Fomivirsen (Vitravene)	Intravitreal		Cytomegalovirus (CMV) retinitis in immunocompromised patients	CMV IE-2 mRNA	1998 (FDA), 1999 (EMA) *

The present document enumerates RNA therapeutics for which approval has been granted by the FDA and/or the European Medicines Agency. The listing is arranged in the reverse chronological order of the most recent approval. Abbreviations; 6MWT, 6 min Walk Test; AE, adverse event; aPTT, Thromboplastin Time; AEs, adverse events; ASO, antisense oligonucleotide; CVD, cardiovascular disease; ds, double-stranded; ECG, Electrocardiogram; FDA; Food and Drugs Administration; GalNAc, *N*-acetylgalactosamine; gen, generation; intravenous, iv; INR, International Normalized Ratio; LDL-C, low-density lipoprotein cholesterol; mNIS, modified Neuropathy Impairment Score PMO; phosphoroamidate morpholino oligomer; PT, phosphothiorate; PTT, Partial Thromboplastin Time; QoL-DN, Quality of Life Diabetic Neuropathy; SAE, adverse event; SC subcutaneously; siRNA, small interfering RNA. * Due to the development of effective antiretroviral therapies, marketing was discontinued in 2002.

**Table 3:** Clinical studies discontinued.

Study Identifier-Acronym/N	Patient (n)/ Treatment	Type	Delivery Method	Target Location	Disease	Target Gene and Pathway	Stady Phase/ Translation Limitation.
Non-RCT multicenter USA NCT00042679 (11) NCT00034268 (2)	26 Aprinocarsen (ISIS 3521, LY900003)	ASO	IV	Tumor	Non-small cell lung cancer Low-grade follicular lymphoma B-cell small lymphocytic lymphoma	Protein kinase Cα mRNA	Phase II No increase in clinical effectiveness
Non-RCT multicenter (66) NCT00003892	22 ISIS 5132 (CGP 69846 A)	ASO	IV	Tumor	Breast cancer, ovarian cancer	*Raf* mRNA	Phase II No increase in clinical effectiveness
RCT multicenter (31) NCT00048321	160 ISIS 104838	ASO	Oral	Joints	Rheumatoid arthritis	TNF mRNA	Phase II Company decision related to cost and competition.
RCT multicenter (51) NCT01445899	258 PF-4523655 (PF-655) Ranibizumab	siRNA	Intravitreal	Eye	Age-related macular degeneration, diabetic macular edema	DNA damage- inducible transcript 4 (*DDIT4*) mRNA	Phase II No increase in clinical effectiveness versus the existing standard of treatment.
RCT multicenter (10) NCT01414101 EudraCT#	51 ISIS 329993 (ISIS-CRPRx)	ASO	SC or intraperitoneal	Heart or joints	Paroxysmal atrial fibrillation, rheumatoid arthritis	C-reactive protein (*CRP*) mRNA	Phase II Despite decreasing CRP mRNA, clinical effectiveness was insufficient.
Interventional (3) NCT00357747	10 AEG35156 (AEG 161, GEM 640)	ASO	IV	Tumor	Various malignancies	X-linked inhibitor of apoptosis (*XIAP*) mRNA	Phase I It is ineffective and increases the risk of neuropathy.
Interventional (15) NCT00138658	85 Custirsen (ISIS 112989, OGX-011, TV-1011)	ASO	IV	Tumor	Prostate cancer, breast cancer	Clusterin (*CLU*) mRNA	Phase III Trials did not meet primary endpoints, showing that it is ineffective.
RCT multicenter COBALT (60) NCT00499590	338 Bevasiranib (Cand5) Ranibizumab	siRNA	Intravitreal	Eye	Age-related macular degeneration, diabetic macular edema	Vascular endothelial growth factor (*VEGF*) mRNA	Phase III TLR3 stimulation has not been clinically effective.
Interventional (1) NCT00091078	96 Oblimersen sodium (G3139, Genasense)	ASO	SC	Tumor	Various malignancies	*BCL2* mRNA	Phase II Insufficient delivery led to ineffective treatment and failed to meet primary endpoints.
Interventional (2) NCT00363714	26 AGN 211745 (AGN-745, siRNA-027)	siRNA	Intravitreal	Eye	Age-related macular degeneration, choroidal neovascularization	Vascular endothelial growth factor receptor 1 (*VEGFR1*) mRNA	Phase III The clinical effectiveness of TLR3 stimulation, which is independent of sequence, has not been demonstrated.
Interventional (1) (NCT00927459)	23 PRO-040201 (TKM-ApoB, ApoB SNALP)	siRNA	IV	Liver	Hypercholesterolaemia	Apolipoprotein B (*APOB*) mRNA	Phase I It may stimulate the immune system and cause flu-like symptoms
Interventional (10) NCT01829971	155 MRX34	miRNA mimic	IV or intratumour	Intravenous or intratumour	Primary liver cancer, advanced or metastatic cancer with or without liver involvement, hematological malignancies	miR-34a targetome	Phase I Adverse immune-related side effects
RCT multicenter (2) EudraCT, number 2013-002978-49.	32 RG-101	AntimiR	SC	Liver	Hepatitis C infection	miR-122	Phase I Adverse immune-related side effects
Interventional Non-RCT(19) NCT02580552	66 χCobomarsen (MRG-106)	AntimiR	SC or IV	lood or lymphoid organs	Various lymphomas	miR-155	Phase I Company takes a separate decisive action unrelated to either safety or effectiveness.
RCT multicenter (22) NCT03907072	6 χSuvodirsen (WVE-210201)	ASO	IV	Muscle	Duchenne muscular dystrophy	Dystrophin (*DMD*) pre-mRNA splicing (exon 51 skipping)	Phase II; Phase III; The treatment’s clinical effectiveness was not demonstrated, and it did not enhance dystrophin production.
RCT multicenter (35) NCT02947867 EudraCT Number 2014-000239-18	333 χAganirsen (GS-101)	ASO	Topical	Eye	Ischaemic central retinal vein occlusion, neovascular glaucoma	Insulin receptor substrate 1 (*IRS1*) mRNA	Phase II; Phase III; Problems associated with stability of the product composition.
RCT multicenter (2) NCT02795325	41 χDCR-PH1	siRNA	IV	Liver	Primary hyperoxaluria type 1 (PH1)	Lactate dehydrogenase A (*LDHA*) mRNA	Phase I Research has centered on a specific type of conjugation called GalNAc, and more specifically, DCR-PHXC.
Interventional (6) NCT02110563	50 χDCR-MYC (DCR-M1711)	siRNA	IV	Tumor	Advanced solid tumors, multiple myeloma, lymphoma	*MYC* mRNA	Phase I Reducing MYC does not result in clinical improvement.

Discontinued medications are reported. Abbreviations used in this text include AntimiR (anti-microRNA), ASO (antisense oligonucleotide), GalNac (N-acetylgalactosamine), gen (generation), IV, intravenous; LNA (locked nucleic acid), PT (phosphothiorate), siRNA (small interfering RNA), SC, subcutaneous; TLR3 (Toll-like receptor 3).

**Table 4:** RNA therapeutic products in phase II or III clinical testing.

Treatment/Biopharmaceutical/ Sponsor	Type	Amendment and Product Delivery	Method of Distribution	Target Site	Ilness	Focus Gene and Pathway	Phase/Study Type and Identifier
RG-125 (AZD4076) AstraZeneca (UK)	AntimiR-103/107	GalNAc-conjugated antagomiR	SC	Liver	Type II diabetes, nonalcoholic fatty liver disease.	miR-103/107	I/II non-RCT NCT04120493
Prexigebersen (BP1001-A) Bio-Path Holdings (USA)	ASO	Liposomal	IV	Blood and/or immune cells	Acute myeloid leukemia, chronic myeloid leukemia	*GRB2* mRNA	II NCT01159028 (non-RCT) NCT04196257 (non-RCT) NCT02781883 (non-RCT)
WVE-120102 Wave Life Sciences Ltd. (USA)	ASO (allele- selectiv)	Stereopure ASO	Intrathecal	Brain	Huntington disease	U-variant of SNP rs362331 (SNP2) in *HTT* mRNA	I/II NCT03225846 (RCT) NCT04617860 (non-RCT)
siG12D-LODER Silenseed Ltd. (Israel)	siRNA	Biodegradable polymeric matrix (PLGA)	Intratumoral	Tumor	Advanced pancreatic cancer	G12D-mutated *KRAS* mRNA	II NCT01188785 (RCT) NCT01676259 (RCT)
rAAV5-miHTT (AMT-130) UniQure Biopharma B.V. (The Netherlands)	Pri-miR-451 backbone	Adeno-associated viral vector (AAV5)	Intrastriatal	Brain	Huntington disease	Huntington (*HTT*) mRNA	II NCT04120493 (non-RCT)
Remlarsen (MRG-201) miRagen Therapeutics, Inc. (USA)	miR-29 mimic	Cholesterol conjugated	ID	Skin	Keloid (pathological fibrosis)	miR-29 targetome	II NCT03601052 (Animal model)
Miravirsen (SPC3649) Santaris Pharma A/S (Denmark)	AntimiR-122	PS-β-d-oxy-LNA gapmer ODN	SC	Liver	Hepatitis C virus infection	miR-122	II NCT01727934 (non-RCT) NCT01872936 (non-RCT) NCT01200420 (RCT)
Olpasiran (AMG 890, ARO-LPA) Amgen (Responsible Party) (USA)	siRNA	GalNAc conjugated	SC	Liver	Cardiovascular disease	Apolipoprotein A (*LPA*) mRNA	II NCT04270760 (RCT)
Vupanorsen (AKCEA-ANGPTL3-LRx, ISIS 70380) Akcea Therapeutics (USA)	ASO	GalNAc conjugated	SC	Liver	Dyslipidaemias, hyperlipidaemias, hyperlipoprotein- aemias	Angiopoietin- like 3 (*ANGPTL3*) mRNA	II NCT03360747 (non-RCT) NCT03371355 (RCT) NCT04516291 (RCT)
Danvatirsen (IONIS-STAT3-2.5Rx, AZD9150) MedImmune LLC * (The Netherlands)	ASO	GalNAc conjugated	IV	Tumor	Metastatic NSCLC, resectable early-stage NSCLC *, pancreatic cancer, mismatch repair-deficient colorectal cancer	*STAT3* mRNA	II NCT03794544 * (RCT) NCT0298357 (aniaml model)
Cemdisiran (ALN-CC5) Alnylam Pharmaceuticals (France)	siRNA	GalNAc conjugated	SC	Blood	Paroxysmal nocturnal haemoglobinuria, IgA nephropathy, Berger disease, glomerulonephritis	Complement 5 mRNA	II NCT02352493 (RCT) NCT03841448 (RCT) NCT03999840 (RCT)
BMT 101 (cp-asiRNA) HUGEL (Republic of Korea)	Cell-penetrating asymmetrical siRNA	Carrier-free	ID	Skin	Hypertrophic scar	Connective tissue growth factor (*CTGF*) mRNA	II NCT04012099 (non-RCT)
Apatorsen (OGX-427) SCRI Development Innovations, LLC (USA) Achieve Life Sciences Queen Mary University of London (UK)	ASO	2′-O-MOE-PTO gapmer	IV	Tumor	Squamous cell lung cancer, non-squamous NSCLC, urological neoplasms, metastatic bladder cancer, urinary tract neoplasms, castration-resistant prostate cancer	*HSP27* mRNA	II NCT01120470 (RCT) NCT01454089 (RCT) NCT01829113 (RCT) NCT02423590 (RCT)
Bamosiran (SYL040012) Sylentis, S.A. (Spain)	siRNA	Carrier-free	Topical	Eye	Ocular hypertension, glaucoma	β-Adrenergic receptor 2 (*ADRB2*) mRNA	II NCT01227291 (non-RCT) NCT01739244 (RCT) NCT02250612 (RCT)
Donidalorsen (IONIS-PKK-LRx, ISIS 721744) Ionis Pharmaceuticals, Inc. (USA)	ASO	GalNAc- conjugated PS-2′-MOE ODN	SC	Liver	Hereditary angio-edema, COVID-19	Prekallikrein (*PKK*) mRNA	II NCT04030598 (RCT) NCT04307381 (non-RCT)
Sepofarsen (QR-110) Laboratoires Thea (France)	ASO	Chemically modified	Intravitreal	Eye	Leber congenital amaurosis type 10 (LCA10) is a hereditary or congenital eye disease that can cause blindness and vision and sensation disorders. It may also present with neurological manifestations. LCA10 falls under the category of eye diseases.	c.2991 + 1655A > G-mutated CEP290, pre-mRNA splicing	II/III NCT03140969 (RCT) NCT03913143 (RCT) EudraCT 2018-003501-25 (RCT) NCT03913130 (RCT)
Tominersen (RO7234292, HTT ASO, IONIS-HTTRx, ISIS-443139, ISIS-HTTRx, RG 6042) Ionis Pharmaceuticals, Inc. (USA)	ASO (allele- nonselective)	PS-2′-MOE gapmer	Intrathecal	Brain	Huntington disease	*HTT* mRNA	III NCT03342053 (RCT) NCT03761849 (RCT) NCT03842969 (RCT)
AKCEA- TTR-LRx Ionis Pharmaceuticals, Inc. (USA)	ASO	GalNAc conjugated	SC	Liver	Hereditary transthyretin-mediated amyloid polyneuropathy	*Transthyretin (TTR)* mRNA	II; III NCT03728634 (RCT) NCT04136184 (RCT) NCT0413617 (RCT)
Alicaforsen (ISIS 2302) Ionis Pharmaceuticals, Inc. (USA) Atlantic Pharmaceuticals Ltd. (Hong Kong)	ASO	Phosphorothioate-modified	Oral	Intestine	Crohn’s disease	*ICAM1* mRNA	II/III NCT00063830 (RCT) NCT00063414 (RCT) NCT00048113 (RCT) NCT02525523 (RCT)
Nedosiran (DCR-PHXC) Novo Nordisk A/S (Denmark)	siRNA	GalNAc conjugated	SC	Liver	Primary hyperoxaluria type 1 and type 2 are kidney and urological diseases characterized by excessive oxalate production.	Lactate dehydrogenase A enzyme *(LDHA)* mRNA	II; III NCT04580420 (non-RCT) NCT03847909 (RCT) NCT04042402 (non-RCT)
Tivanisiran (SYL1001) Sylentis, S.A. (Spain)	siRNA	Carrier-free	Topical	Eye	Dry eye disease	TRPV1 is a member of the transient receptor potential cation channel subfamily V.	II; III NCT01776658 (RCT) NCT02455999 (RCT) NCT03108664 (RCT)
Pelacarsen (AKCEA-APO(a)-LRx, TQJ230) Akcea Therapeutics (USA) Novartis Pharmaceuticals (France)	siRNA	GalNAc conjugated	SC	Liver	Hyperlipo- proteinaemia	Apolipoprotein A mRNA	II; III NCT03070782 (RCT) NCT04023552 (RCT)

As outlined in the table, the various phases of experimentation for miRNAs are shown alongside their corresponding levels of study progression. This is reflected in an increased focus on the study phase. Abbreviations used in this text include: ASO, antisense oligonucleotide; GalNAc, *N*-acetylgalactosamine; ID, intradermal; IV; intravenous; LNA, locked nucleic acid; LODER, local drug eluter; NSCLC, non-small cell lung cancer; SC; subcutaneous; siRNA, small interfering RNA; SNP, single nucleotide polymorphism. * Neoadjuvant Durvalumab Alone or in Combination With Novel Agents in Resectable Non-Small Cell Lung Cancer.

**Table 5:** Completed or conducting ongoing clinical trials of miRNAs with therapeutic potential.

Active Principle/ Therapeutic Drug Name	Indication	Identifier/Clinical Phase	Study Type/Enrollment Patients (N)	Findings	Corporate Sponsor	Related Cardiovascular Studies
miR-132-3p inhibitor (CDR132L)	Stable heart failure	NCT04045405 Phase I NCT05350969 Phase II	Single center RCT (28) Multicenter RCT (294)	The pig model shows good pharmacokinetics, safety and tolerability. High clinical potential for the antimiR-132 treatment [28].	Cardior Pharmaceuticals	[15,28,31,135]
miR-122-5p inhibitor (miravirsen)	HCV	NCT00688012 Phase I NCT00979927 Phase I NCT01646489 Phase I NCT01200420 EudraCT 2010-019057-17 Phase IIa	Single center RCT (64) Single center RCT (30) Single center non-RCT (5) Multicenter RCT 38	Miravirsen reduced HCV RNA levels in chronic genotype 1 infection, without viral resistance. No long-term safety among 27 patients treated with miravirsen. Larger trials required [180,181,182].	Santaris Pharma	[180,181,182,183]
miR-103/107-3p inhibitor (AZD4076)	T2D with NAFLD T2D with NASH	NCT02826525 Phase I/IIa Halted for strategic reasons NCT02612662 Phase I Halted for strategic reasons	Multicenter RCT (46) Single center RCT (40)	miR-103 programs ECs toward a maladapted phenotype through targeting of lncWDR59, which may promote atherosclerosis [183].	AstraZeneca	[172,173,174,175,176,177,178]
miR-122-5p inhibitor (RG-101)	HCV	EudraCT 2015-004702-42 Phase II EudraCT 2015-001535-21 Phase II EudraCT 2013-002978-49 Phase IIb EudraCT 2016-002069-77 Phase IIb	Multicenter RCT (--) * “” † (--) “” (--) “” (--)	Higher doses required to achieve the SVR rates to enable a single-visit curative regimen [184]. 2 mg/kg or 4 mg/kg well-tolerated and reduced viral load within 4 weeks. Sustained virological response in three patients for 76 weeks [185].	Regulus Therapeutics	[174,175,176,177,178,179]
miR-16-5p mimic (TargomiR)	Malignant pleural mesothelioma	NCT02369198 Phase I	Multicenter non-RCT (27)	TargomiRs support further studies of TargomiRs in combination with chemotherapy or immune checkpoint inhibitors [186].	Asbestos Diseases Research Foundation	
miR-17-5p inhibitor (RGLS4326)	ADPKD	NCT04536688 Phase Ib	Multicenter non-RCT (19)	RGLS4326 is safe in preclinical studies and reduces cyst growth in human and mouse models of PKD. Preclinical characteristics support development for ADPKD [187].	Regulus Therapeutics	[188,189]
miR-155-5p inhibitor cobomarsen (MRG-106)	Cutaneous T-cell lymphoma	NCT02580552 Phase I NCT03713320 Phase II (terminated for strategic reasons)	Multicenter non-RCT (66) Multicenter RCT (37)	Findings support the use of cobomarsen in ABC-DLBCL and other types of lymphoma with elevated miR-155 expression [134].	miRagen Therapeutics (now Viridian Therapeutic)	[32,34]
miR-92a-3p inhibitor (MRG-110; S 95010)	Wound healing CVD	NCT03603431 Phase I NCT03494712 Phase I EUDRA-CT 2017-004180-12 Phase I	Single center RCT(42) Single center RCT(49)	miR-92a targets are derepressed in a cell type-specific manner. Infusion of antimiR-92a efficiently inhibits miR-92a [133].	miRagen Therapeutics (now Viridian Therapeutic)	[45,79,190,191,192,193,194,195]
miR-21-5p inhibitor lademirsen (RG-012)	Alport’s syndrome	NCT02855268 Phase II (ongoing)	Multicenter RCT (37)	antimiR-21 and ACEi therapies is effective on kidney function, pathology and survival in Alport mouse model, supporting the addition of aantimiR21 to the current standard of care (ACEi) [192].	Genzyme/ Sanofi	
miR-29-3p mimic remlarsen (MRG-201)	Keloid scar formation	NCT02603224 Phase I NCT03601052 Phase II	Single center RCT(54) NCT01829971	miR-29b therapy could reduce vascular inflammation [25].	miRagen Therapeutics (now Viridian Therapeutic)	[21,22,23,25]
miR-34a-5p mimic (MRX-34)	Advanced cancer	NCT01829971 Phase I (terminated due to serious adverse effects)	Multicenter non-RCT (155)	MRX34 treatment with dexamethasone premedication well-tolerated showing some clinical activity. The trial stopped early due to severe immune-related AEs [72].	Mirna Therapeutics	[73,186,196,197]

The table summarizes 19 clinical trials that have used microRNA-based therapeutics. Abbreviations used in this text include: ACEi, angiotensin-converting enzyme inhibitor; ADPKD, autosomal dominant polycystic kidney disease; AE, adverse event; T2D, type 2 diabetes; NAFLD, nonalcoholic fatty liver disease; NASH, nonalcoholic steatohepatitis; HCV, hepatitis C virus. Refs. [15,17,22,23,28,31,32,34,73,79,180,181,182,183,184,185,186,187,188,189,190,191]. * N patients non reported; † Multicenter RCT.

**Table 6:** Composition, mode of delivery and dosage schedules of selected synthetic inhibitors or mimics of microRNAs.

Synthetic Molecule φ Ref	Organism	Composition	MoD	Findings	Dosage Schedules
LNA-antimiR-29 [17]	Mouse	Saline	i.v.	MiR-29 causes cardiac problems. In a mouse model, preventing or reducing miR-29 prevents cardiac problems.	20 mg/kg, 1 daily dose for 3 days, starting d1 after surgery.
LNA-antimiR-15b [207]	Mouse	Saline	i.v. via catheter	The use of miR-15 as a therapeutic target for addressing cardiac remodeling and dysfunction in cases of ischemic injury.	Up to 33 mg/kg, 1 dose 3 days after AngII infusion.
LNA-antimiR-26a or miR-26a mimic [208]	Mouse	Matrigel	s.c.	miR-26a regulates EC angiogenic responses via BMP/SMAD1. Its expression could offer a new target for ischemic disease treatment.	1 × 10^6^ cells/mL Matrigel transfection: 30–100 nM oligonucleotide/5 × 10^4^ cells
LNA-antimiR-15 [209]	Mouse	Saline	s.c.	The miR-15 family regulates cardiac hypertrophy and fibrosis by inhibiting the TGFβ pathway.	2 doses with 5 mg/kg each (2–3 days before TAC, 3–4 days after)
LNA-antimiR-26a [208]	Mouse	Not candidate	i.v.	In zebrafish, miR-26a overexpression inhibited formation of the caudal vein plexus, a bone morphogenic protein-responsive process, an effect rescued by ectopic SMAD1 expression.	24 mg/kg, 1 dose 24 h before MI
LNA-antimiR-15b [209]	Pig	Saline	i.v.	Inhibition of miR-15b by subcutaneous injections of LNA-based antimiRs in C57BL/6 mice subjected to transverse aorta constriction led to exacerbated fibrosis and, to a lesser extent, also hypertrophy.	Up to 3.3 mg/kg
LNA-antimiR-22 [210]	Mouse	Hydrogel	Perivascular	miR-22 and EVI1 are novel regulators of VSMC function during neointima hyperplasia, offering a new therapeutic opportunity for treating vascular diseases.	2.5 nmol Injection concomitant with surgery
LNA-antimiR-21 [18]	Pig	Saline	i.v.	miR-21 is a target for heart failure treatment and show that microRNA therapy works in cardiovascular diseases.	10 mg each on d5 and d19 after MI
Antagomirs					
Antagomir-199b [38]	Mouse	Saline	i.p.	In vivo silencing of miR-199b-5p in MI-induced cardiac remodeling using an antagomir to inhibit endogenous miR-199b-5p effectively suppresses cardiac miR-199b-5p expression, attenuating cardiac dysfunction and dilation following MI.	0.05–80 mg/kg
Antagomir-33 [212]	Mouse	Saline	i.p.	Anti-miR33 treatment raises HDL and promotes regression of atherosclerosis. Promising strategy for treating atherosclerotic vascular disease.	80 mg/kg, 1 daily dose for 3 days, starting day 1 after surgery
Antagomir-21 [18]	Mouse	Saline	i.v. via catheter	miR-21 impact on global cardiac structure and function. miR-21 levels are selectively increased in fibroblasts of the failing heart, leading to enhanced ERK-MAP kinase activity through the inhibition of sprouty homologue 1 (Spry1).	80 mg/kg, 1 daily dose for 2 days, starting d1 or d21 after surgery.
Antagomir-29b [17]	Mouse	Saline	i.p.	Targeted deletion of miR-29 in cardiac myocytes in vivo prevents cardiac hypertrophy and fibrosis. The function of miR-29 in cardiac myocytes is more important than that in non-myocyte cell types.	80 mg/kg, 1 daily dose for 2 days, starting d1 or d21 after surgery
Antagomir-146 [213]	Mouse	Saline	Not indicated	The relationship between miR-146a and AMPK provides new insight into the role of miRNAs in NAD+/SIRT regulation and the potential for interventions to prevent aging and age-related diseases.	8 mg/kg d2 before delivery and d1, d3 and d7 after surgery

Displays the therapeutic dosage and composition administered in the animal model. Abbreviations; MoD, mode of delivery; AngII, angiotensin II; i.v., intravenous; s.c., subcutaneous; i.p., intraperitoneal.

## Data Availability

No new data were created or analyzed in this study. Data sharing is not applicable to this article.

## Abbreviations

ACS	acute coronary syndrome.
AMI	acute myocardial infarction.
ASOs	antisense oligonucleotides.
antimiRs	anti-microRNAs.
CAD	coronary artery disease.
circRNAs	circular RNAs.
long nc RNAs	long non-coding RNAs.
miRNA	microRNAs.
ncRNA	non-coding ribosomal RNA.
SA	stable angina.
shRNAs	short hairpin RNAs.
siRNAs	small interfering RNAs.
UA	unstable angina.
